# Effects of Sex and Diet on Gut Microbiota of Farmland-Dependent Wintering Birds

**DOI:** 10.3389/fmicb.2020.587873

**Published:** 2020-11-12

**Authors:** Gang Liu, Derong Meng, Minghao Gong, Huixin Li, Wanyu Wen, Yuhang Wang, Jingying Zhou

**Affiliations:** ^1^Research Institute of Wetland, Chinese Academy of Forestry, Beijing, China; ^2^Beijing Key Laboratory of Wetland Services and Restoration, Beijing, China; ^3^Biology Department of Cangzhou Normal College, Cangzhou, China; ^4^Tumuji National Nature Reserve, Inner Mongolia, China

**Keywords:** Great Bustard, farmland bird, gut microbiota, diet, sex, microbiota diversity

## Abstract

Gut microbiota plays an important role for bird biological and ecological properties, and sex and diet may be important intrinsic and extrinsic factors influencing gut microbial communities. However, sex difference of gut microbiota has been rarely investigated in free-living birds, and it remains unclear how sex and diet interactively affect avian gut microbiota composition and diversity, particularly under natural conditions. Here we used non-invasive molecular sexing technique to sex the fecal samples collected from two wintering sites of Great Bustard, which is the most sexually dimorphic among birds, as well as a typical farmland-dependent wintering bird. High-throughput sequencing of 16S was applied to identify the gut microbiota communities for both sexes under two diets (wheat_corn and rice_peanut). The results showed that 9.74% of common microbiota taxa was shared among four groups (sex vs. diet), revealing the conservatism of gut microbiota. Microbiota diversity, composition and abundance varied on different diets for male and female Great Bustards, suggesting that the gut microbiota was interactively influenced by both sex and diet. Under the wheat_corn diet, females had higher abundances of the phylum Verrucomicrobia than males, but lower Bacteroidetes and Firmicutes compared to males; meanwhile, the microbiota diversity and evenness were higher for males than females. In contrast, under the rice_peanut diet, females were more colonized by the phylum Firmicutes than males, but less by the phylum Bacteroidetes; while males had lower microbiota diversity and evenness than females. This study investigated the impacts of sex and diet on microbiota of Great Bustards, and highlights the need of new studies, perhaps with the same methodology, taking into account bird ages, flock size, breeding or health status, which will contribute to the understanding of ecology and conservation of this vulnerable species.

## Introduction

Microbiota communities present in the gut contribute to impact behavior, nutrition, metabolism, immunity, endocrinology and other biological properties of the vertebrate host (Cani et al., 2013; Gribble and Reimann, 2019; Glowska et al., 2020; Knutie, 2020; Taylor et al., 2020). Gut microbiota profiles are shaped by intrinsic factors such as genetic background, sex and age, and also determined by extrinsic factors including geographical region, habitat type, and seasonality (Carmody et al., 2015; Hu et al., 2017; Grond et al., 2018). The environment-induced microbiota differences also correlate with diet availability and variation (Amato et al., 2015). Most of previous studies focus on microbiota characterizations of mammalian species, domestic poultry, or laboratory animals (Bolnick et al., 2014; Org et al., 2016). Under controlled conditions, artificial breeding and captive environments are likely to make the gut microbiota differing from that under natural conditions.

Sex differences in microbiota composition are well-characterized in mice and humans (Org et al., 2016; Kim et al., 2020), although the results are inconsistent across studies. Studies of sex differences in gut microbiota mainly focus on domesticated birds, such as chickens (Kers et al., 2018). Birds possibly have a higher reliance on gut microbiota for digestive function due to their lack of initial mechanical digestion (Grond et al., 2018). To our knowledge, sex difference of gut microbiota in free-living birds has been rarely investigated (Kreisinger et al., 2015), because the difficulty of sampling both sexes in the wild may be one of challenges hindering the microbiota study in wild birds. In addition, diet has currently been found to interfere with sex impacts regarding microbiota profiles (Bolnick et al., 2014; Bridgewater et al., 2017). There are several evidence supporting that the avian gut microbiota changes with the shift of feeding habits (Grond et al., 2018; Risely et al., 2018; Wang et al., 2018; Wu et al., 2018; Davidson et al., 2020). However, less is known about the interacting effects of sex and diet on avian gut microbiota under natural conditions.

The Great Bustard (*Otis tarda*), a globally threatened bird, has two subspecies, western Great Bustard (*O.t.tarda*) and eastern Great Bustard (*O.t.dybowskii*). While populations of western Great Bustard are over 50,000 (Alonso and Palacín, 2010), only 1,456–2,187 eastern Great Bustards remain (Liu et al., 2017). As one of the world’s heaviest flying bird (Martín et al., 2007), Great Bustard shows the highest sexual size dimorphism (SSD) among birds (Alonso et al., 2009). As opportunistic foragers, male Great Bustards have higher dietary diversity than females, except during the post-mating season, suggesting the species’ extreme SSD along with the distinct reproductive role of each sex might explain the trophic niche divergence and sex-specific differences in diet and foraging (Bravo et al., 2012, 2014, 2016, 2019; Bautista et al., 2017).

Eastern populations of Great Bustards migrate to China from breeding grounds located in Mongolia, Russian South Siberia and northeastern China (Kessler et al., 2013), but western Great Bustards migrate to other sites or do not migrate at all. Eastern Great Bustards rely heavily on farmlands during the wintering season by consuming the agricultural food and roosting in the farmland habitats (Yu et al., 2008; Mi et al., 2016; Liu et al., 2018), and agricultural fields accounted for 74% of wintering areas in China (Mi et al., 2016). Diet varied depending on food availability in different wintering regions for Great Bustards (Liu et al., 2018). Selection of wheat habitats was found to differ between sexes of Great Bustards (Gooch et al., 2015). Migrating birds are able to adapt to the local environment by regulating their gut microbiota in response to the diet variations, including greylag geese (*Anser anser*) and ruddy shelducks (*Tadorna ferruginea*) (Wang et al., 2018), swan geese (*Anser cygnoides*) (Wu et al., 2018), and great tits (*Parus major*) (Davidson et al., 2020), however, how microbiota differs between sexes on different diets is unexplored previously. Due to the above characteristics, farmland-dependent wintering Great Bustards are a good candidate to investigate the effects of sex and diet on the gut microbiota.

The aim of this study was to explore the gut microbiota difference between male and female Great Bustards wintering at different sites with variable diet. Thus, we hypothesized, (1) the gut microbiota composition is different between females and males; (2) Great Bustards can alter gut microbiota according to diet, as in other bird species; while (3) sex and diet has an interactive effect on the gut microbiota. The observed differences due to either sex or diet highlighted the importance of shaping the gut microbiota in response to the environment.

## Materials and Methods

### Study Sites and Samples Collection

**FIGURE 1:**
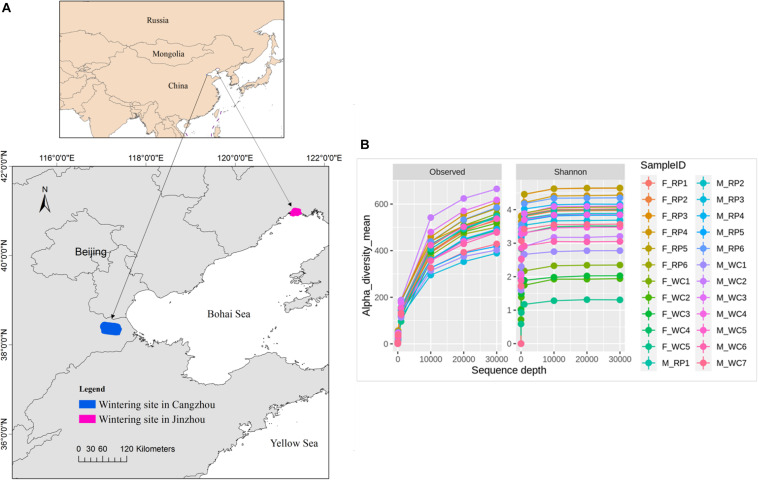
**(A)** Fecal sampling sites of Asian Great Bustard in wintering site in Cangzhou (blue), Hebei province and Jinzhou (red), Liaoning province. **(B)** The sequencing depth and rarefaction curve for each sampling individual. F_RP: females with Rice_Peanut diet, M_RP: males with Rice_Peanut diet, F_WC: females with Wheat_Corn diet and M_WC: males with Wheat_Corn diet.

Fecal samples of Great Bustard were collected from two wintering regions in Jinzhou, Liaoning Province and in Cangzhou, Hebei province in December, 2017 (Figure 1A). Based on the surveying report from our team, 52 individuals fed and roosted in the rice farmlands and peanut farmlands in Jinzhou wintering region, and 3 flocks were observed with a flock size of 27, 15, and 10, respectively. For Jinzhou population, we classified the diet type of Great Bustards as Rice_Peanut (RP). In Cangzhou wintering region, about 200 birds and overwintered here every year (Wu et al., 2011), and several flocks distributed widely in Cangzhou in comparison to Jinzhou. The flock structure (i.e., sex of each flock) was not recorded during the sampling in the wild, and the association within the same flock was not taken into account either. Wheat and corn farmlands were the main foraging areas in Jinzhou, where neither rice nor peanut farmland was found, so the diet type of Great Bustards was classified as wheat_corn (WC). To be noticed, although the wild weeds were also eaten by Great Bustards, agricultural seeds in the harvested grain fields were the dominant and stable food resources (Liu et al., 2018). The fresh fecal samples were collected after the birds flew away, and only fecal samples with a minimum distance interval of five meters were collected in order to avoid recollecting the same individual. Twenty two fecal samples were collected in Jinzhou (diet RP), and 26 in Cangzhou (diet WC). Fecal samples were immediately frozen and stored at −20°C in the field and later stored at −80°C in the lab.

### Molecular Sexing

In order to investigate the sexual difference of gut microbiota in Great Bustard, the fecal samples collected were firstly sex identified using the molecular method. Genomic DNA was extracted using the QIAamp DNA stool minikit (Qiagen, Inc., Valencia, CA, United States). The PCR was performed to amplify the Z-linked CHD fragment using the sexing primers P3 (AGATATTCCGGATCTGATAGTGA) and P2 (TTTCCTAAATCGCTACGTCT) (Martín et al., 2000). The PCR amplication reactions were carried out in a volume of 25 μl containing 2 μl fecal DNA, 2 μl bovine serum albumin (BSA) (Takara, 1 mg/ml), 1 U Amplitaq Gold DNA polymerase (Applied Biosystems, Waltham, MA, United States), 0.3 μM each primer, and 2.5 μl 10 × buffer. Fecal DNA was amplified with an initial denaturing (94°C, 5 min), 30 cycles of denaturation (94°C, 45 s), annealing (55.6°C, 45 s) and elongation (72°C, 45 s), and a final extension step for 10 min at 72°C. By analyzing the sequence, if the fragment was cut into two pieces by *Hae*III restriction enzyme, the fecal sample was female, otherwise male. Due to the low quality of some fecal samples, fecal DNA extraction and PCR failure, finally, for the rice_peanut diet type, six males and six females were identified (M_RP, F_RP, respectively), and for the wheat_corn diet type, seven males and five females were included in the subsequent analysis (M_WC, F_WC, respectively).

### DNA Isolation, Amplification, and Sequencing

Total fecal DNA extraction was performed using the QIAamp DNA stool minikit (Qiagen, Inc., Valencia, CA, United States). Two blank extractions were included to detect cross contamination during DNA isolation process. The bacterial primers 515F (5′-GTGCCAGCMGCCGCGGTAA-3′) and 806R (5′-GGACTACHVGGGTWTCTAAT-3′) were used to amplify the highly variable V3–V4 region of bacterial 16S rRNA (Caporaso et al., 2012). The PCR reactions were carried out in a total of 50 μl volume, using 0.5 μl AmpliTaq Gold DNA Taq polymerase (Applied Biosystems, Waltham, MA, United States) and 3 μl of DNA template. The PCR cycling conditions were as follows: an initial denaturing at 95°C for 5 min; followed by 30 cycles of 95°C for 35 s, 56°C for 30 s, and 72°C for 35 s, and then finished with a final extension at 72°C for 10 min. The PCR products were pooled in equimolar concentrations on a 2% agarose gel, and purified PCR products were sequenced using the Illumina MiSeq sequencing platform (Illumina, United States) at Shanghai Sangon Biotech, Co. Ltd. (Shanghai, China).

### Statistical Analysis

Generated sequencing reads were quality filtered, joined and demultiplexed using the standard operating procedure using QIIME version 2 (Bolyen et al., 2019). Chimeras were identified with the UCHIME algorithm and removed from the dataset. The remaining sequences were clustered into operational taxonomic units (OTUs) with a 97% sequence similarity threshold, and assigned to taxa using the SILVA v132 bacterial taxonomy database (Quast et al., 2013). After taxonomy assignment, OTUs identified as chloroplasts, mitochondria or Archaea were removed from the dataset.

To evaluate the sequencing effectiveness, rarefaction curves were generated based on both the number of observed unique amplicon sequencing variants and the Shannon index for each sample. The R package phyloseq (McMurdie and Holmes, 2013) was used to calculate alpha diversity parameters of Shannon index as an indicator of gut microbiota diversity. Shannon index usually lies between 1.5 and 3.5, rarely surpassing and higher value indicates higher microbiota community diversity (Magurran, 1988). Pielou’s evenness was applied to reflect species equitability, ranging from 0 to 1.0 (Gorelick, 2010). Both the Shannon index and Pielou’s evenness did not follow the normal distribution after a Kolmogorov–Smirnov test (*p* < 0.05), so the non-parametric model Kruskal–Wallis test was conducted in spss version 25 (IBM Corporation, 2017), in order to test the significance including sex and diet as factors. Relative abundance difference between groups was analyzed using non-parametric Mann–Whitney *U*-tests in spss version 25 (IBM Corporation, 2017).

Beta-diversity was examined between samples using the Bray–Curtis dissimilarity index with the aim to measure inter-sample diversity using phyloseq. The distance matrix was analyzed using permutational multivariate analysis of variance with 1000 permutations (Kelly et al., 2015). Statistical significance of sex and diet differences was assessed through a multivariate analysis of variance with permutation using ADONIS tests (Anderson, 2017). Principal coordinates analysis (PCoA) was used to visualize the differences between sexes and diets (Grossmann et al., 2007), and the aim of this analysis was to see whether the gut microbiota profile differs between sexes, and whether it diverges between diets. Based on standard Pearson correlation distance, the hierarchical clustering heatmap was constructed with the Ward clustering algorithm to visualize the relationship between samples at the genera-level taxa using MicrobiomeAnalyst (Chong et al., 2020).

The core microbiota was identified and characterized that was shared by the majority of samples using the microbiota package. The core microbiota was defined as dominant OTUs with a relative abundance of over 1% that were shared among over 50% of the samples. Venn diagrams (Shade and Handelsman, 2012) were plotted with the VennDiagram program (version 1.6.20) to visualize the numbers of core OTUs shared by each group associated with sex and diet.

Differential OTU abundance was analyzed between sexes and between diets using the package DESeq2 (Love et al., 2014). Briefly, the differential abundance and richness analyses in DESeq2 use a generalized linear model of counts following a negative binomial distribution, scaled by a normalization factor that accounts for differences in sequencing depth between samples. Before running DESeq2, the variance-mean dependence estimation was conducted to test whether the variance is greater than the mean, in order to support the reasonability of using DESeq2 (Love et al., 2014). In the models, we included diet and sex as factors. Differential OTU abundances were assessed using the Wald tests, and *p*-values adjusted by the false discovery rate (FDR) using the Benjamin–Hochberg (B–H) method (Matthew, 2017). Log-transformed ratios, instead of absolute count of one taxon, was applied to overcome the limitations inherent to dealing with proportions. The threshold adjp < 0.05 and absolute value of log2FoldChange > 2.0 were used to identify the significant abundant OTU between diets and between sexes. log2FoldChange means the log2 transformation of the abundance estimation fold given an OTU between sexes or between diets. Here a cut of 2 for absolute log2FoldChange is commonly used to reduce the impact of shot noise for low counts, and statistical power can be increased (Anders and Huber, 2010). To validate the results of DESeq2 analysis, Microbiota taxa associated with sex and diet was also tested using LEfSe (Segata et al., 2011) with Galaxy modules provided by the Huttenhower lab, which applies both the factorial Kruskal–Wallis test and linear discriminant analysis (LDA) to estimate effect size (Thorsen et al., 2016). Alpha value for the test among classes was set as 0.05, and threshold on the logarithmic LDA score for discriminative features was set as 2.0, which can be interpreted as the degree of consistent difference in relative abundance between features in the two classes of analyzed microbial communities (Segata et al., 2011).

## Results

### Microbiota Composition and Relative Abundance

The overall abundance was 884,492 across all 24 samples covering all OTUs, with an average sequence reads per sample of 36,583 (Supplementary Figure S1). Total 4,493 OTUs were identified, belonging to 25 phyla, 40 classes, 67 orders, 153 families, 357 genera. The rarefaction curves indicated that the sequencing depth of all samples reached the saturation, meaning the microbiome communities were well-represented (Figure 1B).

**FIGURE 2:**
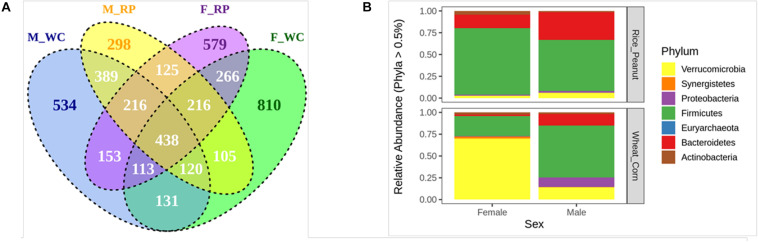
**(A)** The venn diagram of the OTUs in all fecal samples among groups. **(B)** Relative abundance of the microbiota communities at the phylum level for female and male Great Bustards with different diets. OTUs accounting for less than 0.5% of therelative abundance were pruned out prior to plotting. F_RP: females with Rice_Peanut diet, M_RP: males with Rice_Peanut diet, F_WC: females with Wheat_Corn diet and M_WC: males with Wheat_Corn diet.

The Venn diagram showed that 23.33% OTUs were shared by groups M_WC and M_RP, followed by 22.07% OTUs between F_RP and F_WC, 20.01% OTUs in M_RP and F_RP and 17.01% OTUs in M_WC and F_WC (Figure 2A). The percentage of unique OTUs belonging to M_WC, M_RP, F_WC and F_RP was 534 (11.88%), 298 (6.63%), 810 (18.03%), and 579 (12.89%), respectively, based on the flower diagram (Supplementary Figure S2). All four groups shared 438 OTUs (9.74%), including members of the genus *Akkermansia, Alistipes*, *Butyricicoccus*, *Clostridium*, *Bacteroides*, *Faecalibacterium, Flavonifractor*, *Oscillibacter*, *Ruminococcus*, *Subdoligranulum*. Based on the cut-offs for prevalence (50%) and detection (0.1%), 46 core OTUs were most commonly shared among the four sample groups, including the dominate phylum Firmicutes (69.5% OTUs), Bacteroidetes (19.5% OTUs) and Actinobacteria (6.5% OTUs), and the rare phylum Proteobacteria (2.2% OTUs) and Verrucomicrobia (2.2% OTUs).

The compositional differences were reflected based on relative abundances on different sexes or diets. The dominant phyla across all samples were shown as follows (mean percentage of relative abundance ± SE): Firmicutes (55.7% ± 4.9); Verrucomicrobia (22.8% ± 5.8); Bacteroidetes (15.2% ± 2.7); Proteobacteria (4.4% ± 2.8); Synergistetes (3.1% ± 0.9); Actinobacteria (2.8% ± 0.5) (Figure 2B). Bacteroidetes was significantly higher in males than females (*N* = 24, *z* = −2.52, *p* = 0.01), and tended to increase for Rice_Peanut diet compared to Wheat_Corn diet (*N* = 24, *z* = −2.77, *p* = 0.01). In contrast, Actinobacteria was significantly higher in females than males (*N* = 24, *z* = −2.18, *p* = 0.03), but no difference was observed between the diet type (*N* = 24, *z* = −1.02, *p* = 0.31).

### Effects of Sex and Diet on Microbiota Diversity

**FIGURE 3:**
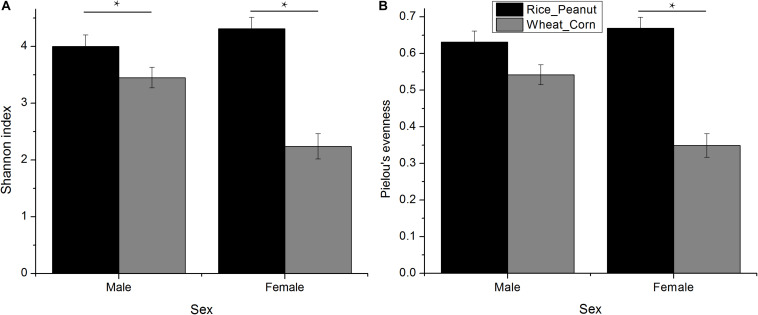
Difference of alpha diversity between groups. **(A)** Shannon diversity indexes calculated for sexes with different diets, and **(B)** Pielou’s evenness for sexes with different diets. Significant difference with asterisks and lines were given, * means *p* < 0.05. The error bar (SE) was shown.

The Kruskal–Wallis test revealed that there was an interaction effect of sex and diet on Shannon index (*H* = 4.01, *p* = 0.005), with males higher than females (*H* = 1.39, *p* = 0.007), and the Rice_Peanut diet higher than the Wheat_Corn diet (*H* = 12.05, *p* < 0.001) (Figure 3A and Supplementary Table S1). The Kruskal–Wallis test indicated that Pielou’s evenness was significantly different between diet (*H* = 12.2, *p* < 0.001), with the Rice_Peanut diet more uniform than the Wheat_Corn diet (Supplementary Table S2), but it was not between sex (*H* = 1.74, *p* = 0.19) (Figure 3B).

**FIGURE 4:**
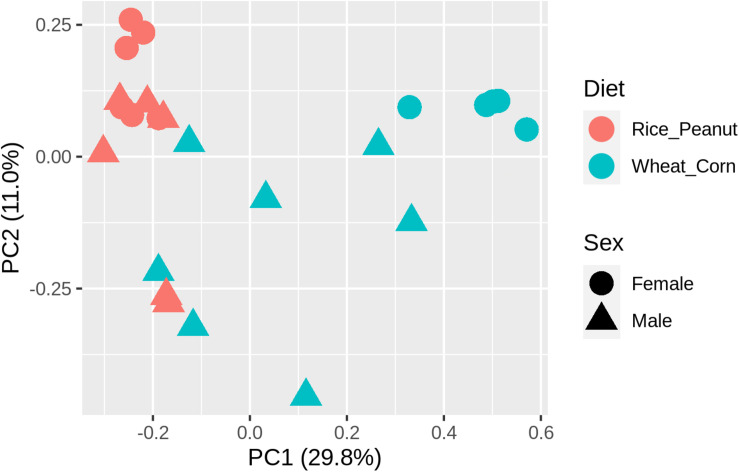
The principal component analysis (PCA) diagram using Bray–Curtis distances on the normalized abundance of OTUs between female and male Great Bustards with different diet.

**FIGURE 5:**
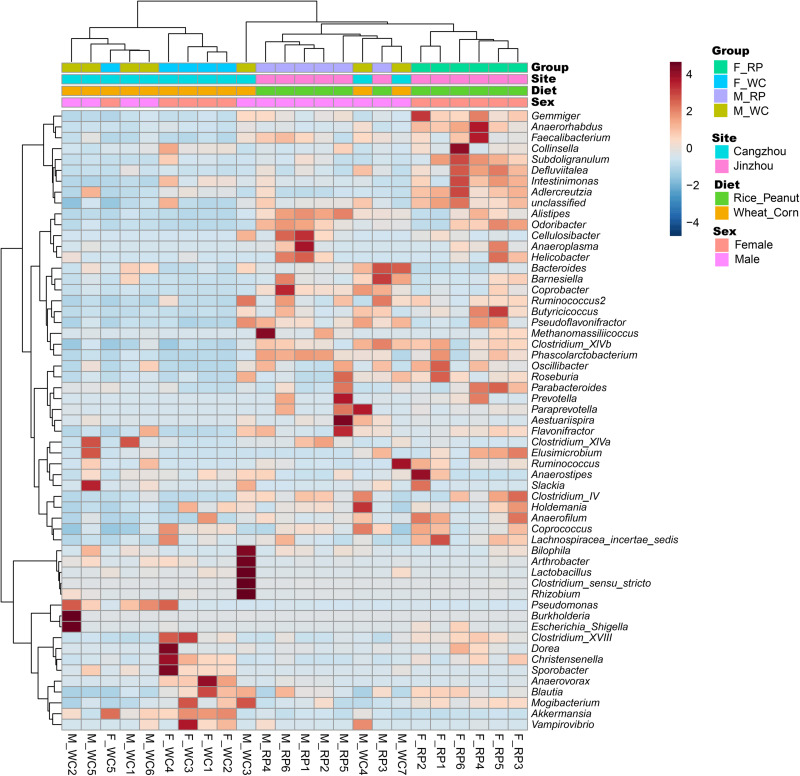
Heatmap showing the relationship between samples based on standard Pearson correlation distance. The tree shown on the left side of the figure depicts hierarchical clustering based on Ward clustering algorithm, and the tree on the top presents samples clustered by the same algorithm. F_RP: females with Rice_Peanut diet, M_RP: males with Rice_Peanut diet, F_WC: females with Wheat_Corn diet and M_WC: males with Wheat_Corn diet.

ADONIS tests showed diet and sex interactively influenced the beta diversity based on Bray–Curtis dissimilarity index (*R*^2^ = 0.35, *p* = 0.012), with significant microbiota difference between diets (*R*^2^ = 0.21, *p* = 0.001), and between sexes (*R*^2^ = 0.11, *p* = 0.019). These results were further supported through the beta PCoA analysis (Figure 4), where males and females were observed to be characterized by a different microbiota community composition, and significant differences were found between diet groups. The first two principle components explained 29.8 and 11.0% of the total variation. Compositional similarity within the genera-level taxa was displayed among individual samples using the heat map, indicating that the samples were categorized into distinguishable dietary groups, and then separated by sex (Figure 5).

### Microbiota Abundance Difference According to Sex and Diet

**FIGURE 6:**
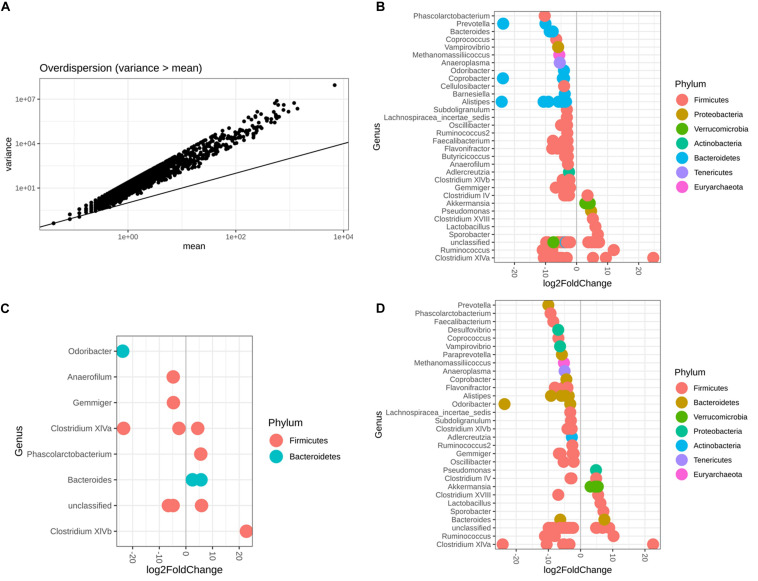
DESeq2 bar plot showing the log2-fold-change of abundance at the Genus and Phylum level. The variance-mean dependence estimation **(A)**, and the OTUs altered in abundance with respect to both sex and diet **(B)**, sex **(C)** and diet **(D)**. log2FoldChange denotes relative differences in abundance between treatments, here log2FoldChange was greater than 2 or less than –2.

It was reasonable to use DESeq2 to conduct differential abundance analysis based on the variance-mean dependence estimation (Figure 6A). With respect to both sex and diet, 119 microbiota OTUs were identified to be differentially abundant by DESeq2, with 68.91% OTUs belonging to the phylum Firmicutes and 21.01% the phylum Bacteroidetes (Figure 6B and Supplementary Table S3). Among 14 identified OTUs associated with sex, 42.85% of them had higher abundance in females than males, belonging to two phyla, Firmicutes and Bacteroidetes (Figure 6C and Supplementary Table S4). In contrast, 84 OTUs were observed as altered in abundance due to diet (Figure 6D and Supplementary Table S5), where 83% OTUs had significantly lower abundance in the Wheat_Corn diet than the Rice_Peanut diet (Figure 6C).

**FIGURE 7:**
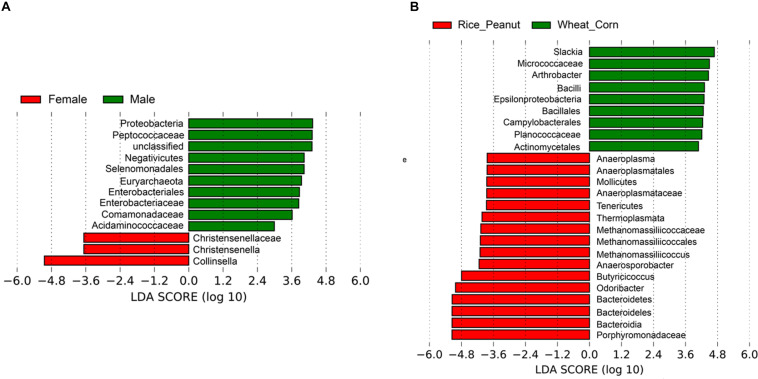
LEfSe analysis showing microbiota OTUs significantly associated with sex and diet. **(A)** Differentially abundant genera related to sex determined using Kruskal–Wallis test (*p* < 0.05) with LDA score > 2.0, **(B)** Differentially abundant genera related to diet determined using Kruskal–Wallis test (*p* < 0.05) with LDA score > 2.0.

Based on the LEfSe results, 12 OTUs were significantly associated with both sexes and 25 taxa were identified as significantly discriminative to diets. Female individuals were associated with three genera-level taxa, Collinsella, Christensenella and Christensenellaceae, while nine genera-level taxa were identified significantly associated within males, including members of Proteobacteria, Peptococcaceae, Negativicutes, Selenomonadales, Euryarchaeota, Enterobacteriaceae, Comamonadaceae, and Acidaminococcaceae (Figure 7A). The LEfSe identified nine genera-level taxa as significantly associated with the Wheat_Corn diet, including *Slackia, Micrococaceae*, *Arthrobacter*, *Bacilli*, *Epsilonproteobacteria*, *Bacillales*, *Campylobacterales*, *Planococcaceae and Actinomycetales*, which were different from the 16 genera-level taxa for the Rice_Peanut diet (Figure 7B).

## Discussion

The results in this study presented the first metagenomic profile of Bustard species, which helps gaining an understanding of the bird-microbiota relationship. Intrinsic factors such as sex effects on the microbiota are difficult to investigate, partly because sampling both sexes or determining the sex of samples might make the study impractical for wild birds, but also because habitat factors such as diet may overshadow sex effects (Elderman et al., 2018). A primary strength of this study is combining the molecular sexing and microbiota study based on fecal samples collected in the wild. Fecal sampling have been regarded as an important non-invasive method for a lot of avian species (Knutie and Gotanda, 2018), including the Great Bustard (Bautista et al., 2013; Liu et al., 2017, 2018).

The microbiota of Great Bustard was dominated by Firmicutes and Bacteroidetes, similar to other avian systems (Hird et al., 2015; Waite and Taylor, 2015; Noguera et al., 2018; Wang et al., 2018). By comparing the OTU composition, 9.74% was shared among all individuals, revealing the host specificity of gut microbiota in Great Bustards. Great Bustards conduct a long distance of 2000 km migration and overwinter across a wide distribution in central and northeast China (Kessler et al., 2013), but the habitat environments changes along with migration might not change the core and dominant microbiota, which is consistent with previous studies that the dominant gut microbiome of the swan goose (*Anser cygnoides*) are conserved after migration (Wu et al., 2018), as well as Swainson’s thrushes (*Catharus ustulatus*) and gray catbirds (*Dumetella carolinensis*) (Lewis et al., 2016). These results suggest that different microbiota sets might perform similar and conservative functions among the sample groups, because the coexistence of these dominants reflects the consequence of long-term selection in the gut environment (Chang et al., 2016).

Microbiota diversity, composition and abundance varied on different diets for male and female Great Bustards, suggesting that the gut microbiota was influenced by both sex and diet. Sex differences in microbiota composition were found both at the phylum level and genera level given the same diet. Under the wheat_corn diet, females had higher abundances of the phylum Verrucomicrobia than males, but lower Bacteroidetes and Firmicutes compared to males (Figure 2); meanwhile, the microbiota diversity and evenness were higher for males than females (Figure 3). In contrast, under the rice_peanut diet, females were more colonized by the phylum Firmicutes than males, but less by the phylum Bacteroidetes; while males had lower microbiota diversity and evenness than females (Figure 3). The microbiota composition was also different between diets for both sexes, which can be supported by the clustering of microbiota by sex and diet (Figures 4, 5). The same pattern of different microbiota communities between sexes was also observed in laboratory animal model avian species, Japanese quail (*Coturnix japonica*) (Borda-Molina et al., 2020). Great Bustard is a bird species of typical sexual dimorphism differing in body size, behavior, physiology and diet (Martín et al., 2007; Palacín et al., 2009; Bravo et al., 2016, 2019), which may pose different selection pressures on the gut microbiota of each sex. For Great Bustards, physiological constraints due to variations in body size cause different food requirements (Bravo et al., 2016, 2019), and thus a microbiota divergence between males and females. This study can support the sexual differences in microbiota, which also deserves to be contrast with previous studies of sexual differences in diet of Great Bustards. Gut microbiota is known to be altered directly by host diet composition (David et al., 2014; Carmody et al., 2015; Knutie, 2020). It has been reported that the diet of Great Bustards varied according to the spatial availability of local food resources (Liu et al., 2018) and by sex (Bravo et al., 2012, 2016, 2019). In addition, Great Bustards heavily depend on agriculture farmlands during the wintering season, with 74% of wintering areas in China consisting of cropland (Mi et al., 2016), and even in the breeding region, they were observed to become resident at breeding sites in Tumuji Nature Reserve in Inner Mongolia because corn and soy fields scattered within and around the reserve provide food resources. Diet-induced microbiota changes was detected in other farmland-dependent wintering birds, such as greylag geese (*Anser anser*) and ruddy shelducks (*Tadorna ferruginea*) (Wang et al., 2018). Our results indicated that altered diet could subsequently change gut microbiota composition and diversity, which supported the hypothesis that varied diet indeed shapes the gut microbiota profile. For migrating birds, food resources and availability change temporarily and spatially, during which the gut microbiota may be locally acquired to help the host to digest and absorb these local prey items, better adapting to the current environment.

Both differential abundance results derived from DESeq2 and LEfSe analysis supported that members of the phyla Firmicutes and Bacteroidetes were associated with sex, while seven phyla (Firmicutesm, Bacteroidetes, Verrucomicrobia, Proteobacteria, Actinobacteria, Tenericutes, Euryarchaeota) were associated with diet (Figure 6). Bacteroidetes and Firmicutes are the most prevalent bacteria phyla in digestive tracts of wild bird species (Grond et al., 2018). Bacteroidetes are considered as symbiotic microbiota essential for the nutrition digestive activity across many animal species (Kohl et al., 2014). Compared with the wheat_corn diet, the abundance of Bacteroidetes increased for the rice_peanut diet and was replaced by Firmicutes (Figure 2B). Members of the phylum Firmicutes are known to have fewer polysaccharide-degrading enzymes than that of the phylum Bacteroidetes (Mahowald et al., 2009), and higher Firmicutes-to-Bacteroidetes ratio could increase the calories uptake efficiency from food (Jones et al., 2019), suggesting that the food composition alteration might change the gut environments by influencing the microbiota communities of Great Bustards. The two wintering sites indeed have geographic differences, which might influence differently the gut microbiota of Great Bustards. However, the effect of geographic location on differences in gut microbiota have been attributed on changes of food availability (San Juan et al., 2020), suggesting changes in microbiota communities could be more sensitive to dietary shifts rather than location (Capunitan et al., 2020). Differences in relative protein, fat, and fiber content may cause changes of microbiota composition and diversity (Clarke et al., 2014). Diet-based shifts in the host gut microbiota might benefit the Great Bustard by helping the host adapt to the local environment, as well as affecting the host health, that should be corroborated with future research.

In conclusion, the dominant microbiota composition remained unchangeable, but the composition, diversity, and abundance were impacted by both sex and diet for Great Bustards. To be noticed, our results highlights the need of new studies, perhaps with the same methodology, taking into account bird ages, flock size, breeding, or health status (parasite). More than 220 farmland bird species in China rely on cultivated land to survive during the winter (Li et al., 2020), however, the increase of agricultural intensity and farmland fragmentation inevitably results in anthropogenic interference with the changes in food webs within farmland habitats, which may pose threats to the farmland species life cycles. Ultimately the host health could be influenced by the diet-induced gut microbiota changes. Future conservation should consider differences in overwintering food resources, which may influence the nutritional quality of both the diet and associated microbiota.

## Data Availability Statement

The original contributions presented in the study are publicly available. This data can be found in NCBI, under accession number PRJNA650248.

## Ethics Statement

The animal study was reviewed and approved by China Wildlife Conservation Associate.

## Author Contributions

GL contributed to the study concept, data collection, data analysis and the writing of the manuscript. DM and JZ collected samples and provided basic data about the Great Bustard wintering sites. MG, HL, WW, and YW contributed to the study concept and revision of the manuscript. All authors contributed to the article and approved the submitted version.

## Conflict of Interest

The authors declare that the research was conducted in the absence of any commercial or financial relationships that could be construed as a potential conflict of interest.
