# Supplementary material for: Effects of Sex and Diet on Gut Microbiota of Farmland-Dependent Wintering Birds
Source: Front Microbiol. 2020 Nov 12;11:587873. doi: 10.3389/fmicb.2020.587873 (PMC7688461; doi:10.3389/fmicb.2020.587873)
Supplement: Supplementary file 1 [file Data_Sheet_1.docx]

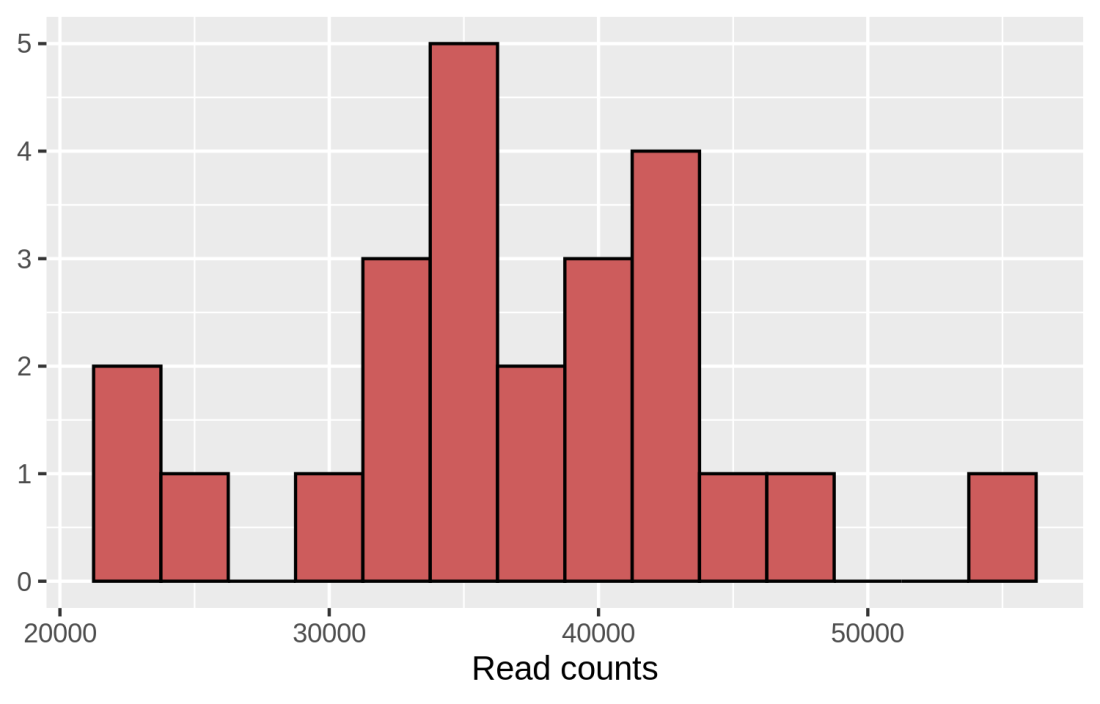


Supplementary Fig. 1 The histogram of distribution of sample sequencing depth.


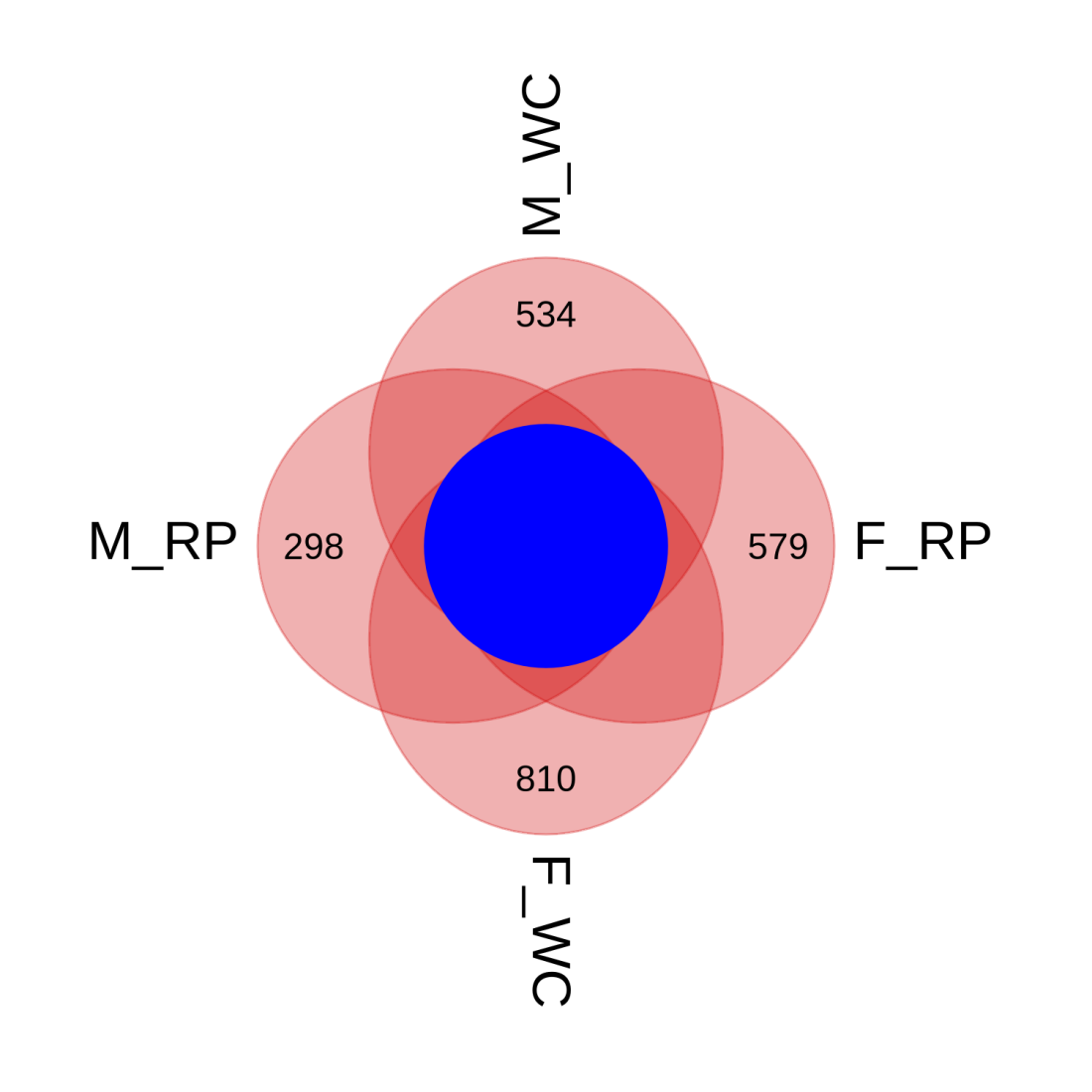


Supplementary Fig. 2 The flower diagram showed the number of unique OTUs belonging to M_WC, M_RP, F_WC and F_RP.
