# Supplementary material for: Effects of Sex and Diet on Gut Microbiota of Farmland-Dependent Wintering Birds
Source: Front Microbiol. 2020 Nov 12;11:587873. doi: 10.3389/fmicb.2020.587873 (PMC7688461; doi:10.3389/fmicb.2020.587873)
Supplement: Supplementary file 2 [file Data_Sheet_2.docx]

Table S1 Non-parametric model Kruskal Wallis test was used to test the Shannon alpha diversity index significance including sex and diet as factors.

| Dependent Variable: Shannon | | | | | |
| --- | --- | --- | --- | --- | --- |
| Source | Type III Sum of Squares | df | H | Sig. |  |
| Sex | 1.185 | 1 | 1.394396 | .007 |  |
| Diet | 10.239 | 1 | 12.05269 | .000 |  |
| Sex * Diet | 3.407 | 1 | 4.009994 | .005 |  |
| Error | 4.709 | 20 |  |  |  |
| Total | 19.539 | 23 |  |  |  |
|  | | | | | |

Table S2 Non-parametric model Kruskal Wallis test was used to test the Pielou evenness index significance including sex and diet as factors.

| **Tests of Between-Subjects Effects** | | | | | |
| --- | --- | --- | --- | --- | --- |
| Dependent Variable:Pielou | | | | | |
| Source | Type III Sum of Squares | df | H | Sig. |  |
| Sex | .035 | 1 | 1.747036 | .19 |  |
| Diet | .248 | 1 | 12.20548 | .000 |  |
| Sex * Diet | .078 | 1 | 3.848843 | .05 |  |
| Error | .105 | 20 |  |  |  |
| Total | .467 | 23 |  |  |  |
|  | | | | | |

Table S3 The DESeq2 results showing the OTUs associated with both sex and diet

| OUT_ID | baseMean | log2FoldChange | lfcSE | stat | pvalue | padj | Phylum | Class | Order | Family | Genus |
| --- | --- | --- | --- | --- | --- | --- | --- | --- | --- | --- | --- |
| Otu163 | 37.64699 | -23.7077 | 2.266168 | -10.4616 | 1.30E-25 | 7.40E-23 | Bacteroidetes | Bacteroidia | Bacteroidales | Porphyromonadaceae | Coprobacter |
| Otu73 | 125.6017 | -10.5327 | 1.182098 | -8.91017 | 5.10E-19 | 1.45E-16 | Firmicutes | Clostridia | Clostridiales | Lachnospiraceae | Clostridium XlVa |
| Otu94 | 59.05266 | 24.70046 | 2.95659 | 8.354372 | 6.58E-17 | 1.25E-14 | Firmicutes | Clostridia | Clostridiales | Lachnospiraceae | Clostridium XlVa |
| Otu52 | 114.4989 | -9.77075 | 1.193573 | -8.18613 | 2.70E-16 | 3.85E-14 | Firmicutes | Clostridia | Clostridiales | Lachnospiraceae | unclassified |
| Otu197 | 21.5876 | -24.1118 | 2.956103 | -8.15661 | 3.45E-16 | 3.93E-14 | Bacteroidetes | Bacteroidia | Bacteroidales | Rikenellaceae | Alistipes |
| Otu8345 | 16.03895 | -23.6273 | 2.956237 | -7.99235 | 1.32E-15 | 1.26E-13 | Bacteroidetes | Bacteroidia | Bacteroidales | Prevotellaceae | Prevotella |
| Otu133 | 77.55505 | -9.2804 | 1.283252 | -7.23194 | 4.76E-13 | 3.88E-11 | Firmicutes | Clostridia | Clostridiales | Ruminococcaceae | unclassified |
| Otu61 | 278.5681 | -5.78619 | 0.842185 | -6.87046 | 6.40E-12 | 4.57E-10 | Bacteroidetes | Bacteroidia | Bacteroidales | Rikenellaceae | Alistipes |
| Otu30 | 565.7969 | -10.8992 | 1.613306 | -6.7558 | 1.42E-11 | 9.01E-10 | Firmicutes | Clostridia | Clostridiales | Ruminococcaceae | Ruminococcus |
| Otu116 | 85.01199 | -7.84688 | 1.194706 | -6.56804 | 5.10E-11 | 2.91E-09 | Firmicutes | Clostridia | Clostridiales | Ruminococcaceae | Flavonifractor |
| Otu429 | 14.17863 | -6.55722 | 1.012527 | -6.4761 | 9.41E-11 | 4.89E-09 | Firmicutes | Clostridia | Clostridiales | Lachnospiraceae | Coprococcus |
| Otu87 | 58.64355 | -10.6184 | 1.79434 | -5.91774 | 3.26E-09 | 1.55E-07 | Bacteroidetes | Bacteroidia | Bacteroidales | Rikenellaceae | Alistipes |
| Otu126 | 122.7446 | -10.0205 | 1.763466 | -5.68228 | 1.33E-08 | 5.84E-07 | Bacteroidetes | Bacteroidia | Bacteroidales | Prevotellaceae | Prevotella |
| Otu45 | 157.6673 | -4.15186 | 0.735604 | -5.64415 | 1.66E-08 | 6.32E-07 | Firmicutes | Clostridia | Clostridiales | Lachnospiraceae | unclassified |
| Otu59 | 259.0558 | -4.07309 | 0.721652 | -5.64412 | 1.66E-08 | 6.32E-07 | Bacteroidetes | Bacteroidia | Bacteroidales | Porphyromonadaceae | Odoribacter |
| Otu332 | 17.62821 | -4.56294 | 0.818193 | -5.57686 | 2.45E-08 | 8.74E-07 | Firmicutes | Clostridia | Clostridiales | Ruminococcaceae | unclassified |
| Otu381 | 13.53454 | -5.81304 | 1.045928 | -5.55779 | 2.73E-08 | 9.18E-07 | Firmicutes | Clostridia | Clostridiales | Lachnospiraceae | unclassified |
| Otu121 | 82.56386 | -4.08326 | 0.740898 | -5.51123 | 3.56E-08 | 1.13E-06 | Firmicutes | Clostridia | Clostridiales | Ruminococcaceae | unclassified |
| Otu177 | 51.85183 | -6.19979 | 1.139449 | -5.44104 | 5.30E-08 | 1.59E-06 | Firmicutes | Clostridia | Clostridiales | Ruminococcaceae | Flavonifractor |
| Otu149 | 50.56541 | -7.60921 | 1.407816 | -5.40498 | 6.48E-08 | 1.85E-06 | Firmicutes | Clostridia | Clostridiales | Ruminococcaceae | Faecalibacterium |
| Otu34 | 355.8909 | -8.65134 | 1.63626 | -5.28727 | 1.24E-07 | 3.38E-06 | Bacteroidetes | Bacteroidia | Bacteroidales | Bacteroidaceae | Bacteroides |
| Otu117 | 63.66358 | -4.53423 | 0.86581 | -5.23698 | 1.63E-07 | 4.24E-06 | Firmicutes | Erysipelotrichia | Erysipelotrichales | Erysipelotrichaceae | unclassified |
| Otu256 | 7.984642 | -6.11909 | 1.170783 | -5.2265 | 1.73E-07 | 4.29E-06 | Firmicutes | Clostridia | Clostridiales | Lachnospiraceae | unclassified |
| Otu251 | 21.68091 | -7.83918 | 1.518564 | -5.16223 | 2.44E-07 | 5.81E-06 | Firmicutes | Clostridia | Clostridiales | Ruminococcaceae | Ruminococcus |
| Otu1 | 58.51526 | 6.06911 | 1.236258 | 4.909259 | 9.14E-07 | 2.09E-05 | Firmicutes | Bacilli | Lactobacillales | Lactobacillaceae | Lactobacillus |
| Otu178 | 74.30913 | -3.73057 | 0.772968 | -4.8263 | 1.39E-06 | 3.05E-05 | Firmicutes | Clostridia | Clostridiales | Lachnospiraceae | Clostridium XlVb |
| Otu78 | 112.4839 | -10.2522 | 2.191033 | -4.67916 | 2.88E-06 | 6.09E-05 | Firmicutes | Negativicutes | Selenomonadales | Acidaminococcaceae | Phascolarctobacterium |
| Otu32 | 1559.438 | -3.07041 | 0.693893 | -4.4249 | 9.65E-06 | 0.000197 | Firmicutes | Clostridia | Clostridiales | Ruminococcaceae | Oscillibacter |
| Otu25 | 503.5811 | -4.03179 | 0.914731 | -4.40762 | 1.05E-05 | 0.000206 | Bacteroidetes | Bacteroidia | Bacteroidales | Porphyromonadaceae | Coprobacter |
| Otu13 | 507.4695 | 11.97294 | 2.73489 | 4.37785 | 1.20E-05 | 0.000227 | Firmicutes | Clostridia | Clostridiales | Ruminococcaceae | Ruminococcus |
| Otu104 | 84.38752 | -4.67684 | 1.069702 | -4.3721 | 1.23E-05 | 0.000227 | Firmicutes | Clostridia | Clostridiales | Lachnospiraceae | Clostridium XlVa |
| Otu216 | 21.86104 | -9.06697 | 2.135093 | -4.24664 | 2.17E-05 | 0.000387 | Bacteroidetes | Bacteroidia | Bacteroidales | Rikenellaceae | Alistipes |
| Otu19 | 806.359 | -3.45111 | 0.816419 | -4.22713 | 2.37E-05 | 0.00041 | Bacteroidetes | Bacteroidia | Bacteroidales | Rikenellaceae | Alistipes |
| Otu203 | 19.30728 | -5.56879 | 1.322902 | -4.20953 | 2.56E-05 | 0.00043 | Euryarchaeota | Thermoplasmata | Methanomassiliicoccales | Methanomassiliicoccaceae | Methanomassiliicoccus |
| Otu124 | 52.75908 | -6.13094 | 1.471901 | -4.16532 | 3.11E-05 | 0.000486 | Proteobacteria | Deltaproteobacteria | Desulfovibrionales | Desulfovibrionaceae | unclassified |
| Otu139 | 112.7042 | -3.25832 | 0.783181 | -4.16037 | 3.18E-05 | 0.000486 | Firmicutes | Clostridia | Clostridiales | Ruminococcaceae | Clostridium IV |
| Otu24579 | 9.027956 | -6.69666 | 1.610123 | -4.1591 | 3.20E-05 | 0.000486 | Firmicutes | Clostridia | Clostridiales | Ruminococcaceae | Gemmiger |
| Otu254 | 12.33003 | -5.81856 | 1.400013 | -4.15608 | 3.24E-05 | 0.000486 | Firmicutes | Clostridia | Clostridiales | Lachnospiraceae | Clostridium XlVa |
| Otu351 | 18.29052 | -3.98821 | 0.963839 | -4.13783 | 3.51E-05 | 0.000512 | Firmicutes | Clostridia | Clostridiales | Ruminococcaceae | Clostridium IV |
| Otu334 | 18.18618 | -4.73774 | 1.14744 | -4.12897 | 3.64E-05 | 0.000512 | Firmicutes | Clostridia | Clostridiales | Lachnospiraceae | unclassified |
| Otu261 | 13.61724 | 6.850319 | 1.659936 | 4.126858 | 3.68E-05 | 0.000512 | Firmicutes | Clostridia | Clostridiales | Ruminococcaceae | Sporobacter |
| Otu200 | 49.97178 | -3.82745 | 0.944785 | -4.05113 | 5.10E-05 | 0.000693 | Firmicutes | Clostridia | Clostridiales | Ruminococcaceae | unclassified |
| Otu159 | 153.8953 | -2.9244 | 0.727448 | -4.02009 | 5.82E-05 | 0.000764 | Firmicutes | Clostridia | Clostridiales | Ruminococcaceae | Faecalibacterium |
| Otu64 | 79.16231 | -4.96376 | 1.235581 | -4.01735 | 5.89E-05 | 0.000764 | Bacteroidetes | Bacteroidia | Bacteroidales | Rikenellaceae | Alistipes |
| Otu48 | 149.6984 | 5.718876 | 1.442079 | 3.965717 | 7.32E-05 | 0.000929 | Firmicutes | Clostridia | Clostridiales | Lachnospiraceae | unclassified |
| Otu22 | 1023.655 | -2.99328 | 0.76203 | -3.92804 | 8.56E-05 | 0.001063 | Firmicutes | Clostridia | Clostridiales | Lachnospiraceae | Ruminococcus2 |
| Otu263 | 21.41545 | -4.75281 | 1.228987 | -3.86726 | 0.00011 | 0.001337 | Firmicutes | Clostridia | Clostridiales | Ruminococcaceae | Flavonifractor |
| Otu28 | 583.9929 | -3.40306 | 0.890402 | -3.82194 | 0.000132 | 0.001575 | Bacteroidetes | Bacteroidia | Bacteroidales | Rikenellaceae | Alistipes |
| Otu97 | 245.5272 | -2.42441 | 0.636226 | -3.81061 | 0.000139 | 0.001615 | Firmicutes | Clostridia | Clostridiales | Ruminococcaceae | Gemmiger |
| Otu105 | 118.8511 | -7.68543 | 2.020777 | -3.80321 | 0.000143 | 0.001631 | Bacteroidetes | Bacteroidia | Bacteroidales | Bacteroidaceae | Bacteroides |
| Otu187 | 67.90087 | -3.45611 | 0.921972 | -3.74861 | 0.000178 | 0.001991 | Firmicutes | Clostridia | Clostridiales | Lachnospiraceae | unclassified |
| Otu1403 | 5.35428 | -5.07096 | 1.386842 | -3.65648 | 0.000256 | 0.002808 | Firmicutes | Clostridia | Clostridiales | Lachnospiraceae | unclassified |
| Otu4 | 9.050463 | 4.667007 | 1.294969 | 3.603952 | 0.000313 | 0.003377 | Proteobacteria | Gammaproteobacteria | Pseudomonadales | Pseudomonadaceae | Pseudomonas |
| Otu0 | 7001.999 | 2.810639 | 0.782049 | 3.593942 | 0.000326 | 0.003444 | Verrucomicrobia | Verrucomicrobiae | Verrucomicrobiales | Verrucomicrobiaceae | Akkermansia |
| Otu299 | 13.71311 | -3.12538 | 0.8737 | -3.57718 | 0.000347 | 0.003589 | Firmicutes | Clostridia | Clostridiales | Lachnospiraceae | Lachnospiracea_incertae_sedis |
| Otu463 | 7.85337 | -4.46223 | 1.248631 | -3.5737 | 0.000352 | 0.003589 | Firmicutes | Clostridia | Clostridiales | Lachnospiraceae | Clostridium XlVb |
| Otu315 | 27.51654 | -3.7045 | 1.047696 | -3.53585 | 0.000406 | 0.004072 | Firmicutes | Clostridia | Clostridiales | Ruminococcaceae | Butyricicoccus |
| Otu55 | 611.5629 | -3.13756 | 0.890255 | -3.52434 | 0.000425 | 0.00418 | Firmicutes | Clostridia | Clostridiales | Ruminococcaceae | Subdoligranulum |
| Otu326 | 10.85104 | -5.40808 | 1.537424 | -3.51762 | 0.000435 | 0.004214 | Tenericutes | Mollicutes | Anaeroplasmatales | Anaeroplasmataceae | Anaeroplasma |
| Otu604 | 10.38218 | -3.72085 | 1.059496 | -3.5119 | 0.000445 | 0.004234 | Firmicutes | Clostridia | Clostridiales | Ruminococcaceae | unclassified |
| Otu136 | 97.44335 | -3.81882 | 1.090032 | -3.5034 | 0.000459 | 0.0043 | Proteobacteria | Betaproteobacteria | Burkholderiales | Sutterellaceae | unclassified |
| Otu305 | 14.57952 | -3.99678 | 1.145065 | -3.49044 | 0.000482 | 0.004441 | Firmicutes | Clostridia | Clostridiales | Lachnospiraceae | Clostridium XlVb |
| Otu16 | 603.6093 | -2.09879 | 0.605866 | -3.46411 | 0.000532 | 0.004822 | Firmicutes | Clostridia | Clostridiales | Ruminococcaceae | Gemmiger |
| Otu72 | 311.5518 | -3.60925 | 1.044492 | -3.45551 | 0.000549 | 0.004831 | Bacteroidetes | Bacteroidia | Bacteroidales | Rikenellaceae | Alistipes |
| Otu239 | 26.96614 | 6.95283 | 2.012309 | 3.455149 | 0.00055 | 0.004831 | Firmicutes | Clostridia | Clostridiales | Lachnospiraceae | unclassified |
| Otu393 | 17.29644 | -2.43575 | 0.707785 | -3.44137 | 0.000579 | 0.005007 | Actinobacteria | Actinobacteria | Coriobacteriales | Coriobacteriaceae | Adlercreutzia |
| Otu132 | 102.9941 | -2.7814 | 0.830646 | -3.34848 | 0.000813 | 0.006837 | Firmicutes | Clostridia | Clostridiales | Ruminococcaceae | Anaerofilum |
| Otu396 | 4.379653 | -5.34611 | 1.596848 | -3.34791 | 0.000814 | 0.006837 | Firmicutes | Clostridia | Clostridiales | Lachnospiraceae | Clostridium XlVa |
| Otu82 | 187.9935 | -3.80919 | 1.145606 | -3.32505 | 0.000884 | 0.007316 | Bacteroidetes | Bacteroidia | Bacteroidales | Porphyromonadaceae | Barnesiella |
| Otu46 | 413.0585 | -2.8328 | 0.856996 | -3.3055 | 0.000948 | 0.007734 | Firmicutes | Clostridia | Clostridiales | Ruminococcaceae | Flavonifractor |
| Otu461 | 4.365728 | 5.242193 | 1.603945 | 3.268312 | 0.001082 | 0.008701 | Firmicutes | Erysipelotrichia | Erysipelotrichales | Erysipelotrichaceae | Clostridium XVIII |
| Otu257 | 49.48871 | -2.29326 | 0.703348 | -3.26049 | 0.001112 | 0.008725 | Firmicutes | Clostridia | Clostridiales | Peptococcaceae 1 | unclassified |
| Otu189 | 72.70162 | -2.80216 | 0.859651 | -3.25965 | 0.001116 | 0.008725 | Firmicutes | Clostridia | Clostridiales | Lachnospiraceae | unclassified |
| Otu222 | 20.18433 | -4.65112 | 1.43413 | -3.24316 | 0.001182 | 0.009121 | Bacteroidetes | Bacteroidia | Bacteroidales | Rikenellaceae | Alistipes |
| Otu594 | 19.23099 | -3.3122 | 1.023418 | -3.23641 | 0.00121 | 0.009215 | Firmicutes | Clostridia | Clostridiales | Ruminococcaceae | unclassified |
| Otu95 | 175.2944 | -2.79964 | 0.872911 | -3.20725 | 0.00134 | 0.010069 | Firmicutes | Clostridia | Clostridiales | Ruminococcaceae | Butyricicoccus |
| Otu205 | 42.22808 | -4.43018 | 1.389116 | -3.18921 | 0.001427 | 0.010456 | Bacteroidetes | Bacteroidia | Bacteroidales | Porphyromonadaceae | Coprobacter |
| Otu470 | 7.022147 | -5.93581 | 1.861419 | -3.18886 | 0.001428 | 0.010456 | Proteobacteria | Deltaproteobacteria | Bdellovibrionales | Bdellovibrionaceae | Vampirovibrio |
| Otu359 | 18.29835 | -4.85231 | 1.532757 | -3.16574 | 0.001547 | 0.011111 | Bacteroidetes | Bacteroidia | Bacteroidales | Rikenellaceae | Alistipes |
| Otu148 | 77.9765 | 9.441514 | 2.984151 | 3.163886 | 0.001557 | 0.011111 | Firmicutes | Clostridia | Clostridiales | Lachnospiraceae | Clostridium XlVa |
| Otu225 | 29.18291 | -4.13816 | 1.310442 | -3.15783 | 0.001589 | 0.011205 | Bacteroidetes | Bacteroidia | Bacteroidales | Porphyromonadaceae | unclassified |
| Otu24416 | 14.36224 | 3.527562 | 1.142842 | 3.086658 | 0.002024 | 0.014054 | Verrucomicrobia | Verrucomicrobiae | Verrucomicrobiales | Verrucomicrobiaceae | Akkermansia |
| Otu167 | 123.5537 | -5.67466 | 1.84008 | -3.08392 | 0.002043 | 0.014054 | Bacteroidetes | Bacteroidia | Bacteroidales | Rikenellaceae | Alistipes |
| Otu70 | 234.9067 | -4.07001 | 1.325304 | -3.071 | 0.002133 | 0.014502 | Firmicutes | Clostridia | Clostridiales | Ruminococcaceae | unclassified |
| Otu408 | 21.12904 | -3.04363 | 0.998435 | -3.0484 | 0.002301 | 0.015455 | Firmicutes | Clostridia | Clostridiales | Lachnospiraceae | unclassified |
| Otu316 | 38.6037 | -3.17368 | 1.044802 | -3.03759 | 0.002385 | 0.015834 | Firmicutes | Clostridia | Clostridiales | Lachnospiraceae | Clostridium XlVa |
| Otu190 | 38.90109 | 3.788673 | 1.250697 | 3.02925 | 0.002452 | 0.015908 | Firmicutes | Erysipelotrichia | Erysipelotrichales | Erysipelotrichaceae | unclassified |
| Otu195 | 61.00863 | 7.235963 | 2.406447 | 3.006907 | 0.002639 | 0.016932 | Firmicutes | Clostridia | Clostridiales | Ruminococcaceae | unclassified |
| Otu8378 | 2.814489 | 4.69732 | 1.582244 | 2.96877 | 0.00299 | 0.01897 | Firmicutes | Clostridia | Clostridiales | Ruminococcaceae | unclassified |
| Otu631 | 4.8605 | -4.7704 | 1.636334 | -2.9153 | 0.003554 | 0.022297 | Firmicutes | Clostridia | Clostridiales | Ruminococcaceae | unclassified |
| Otu295 | 12.53164 | -3.81264 | 1.324732 | -2.87804 | 0.004001 | 0.024763 | Firmicutes | Clostridia | Clostridiales | Ruminococcaceae | unclassified |
| Otu16439 | 16.16962 | -4.6422 | 1.614368 | -2.87555 | 0.004033 | 0.024763 | Firmicutes | Clostridia | Clostridiales | Ruminococcaceae | Gemmiger |
| Otu41 | 285.3748 | -2.78243 | 0.973516 | -2.85812 | 0.004262 | 0.025887 | Firmicutes | Clostridia | Clostridiales | Ruminococcaceae | unclassified |
| Otu56943 | 11.1745 | -3.66184 | 1.28882 | -2.84124 | 0.004494 | 0.027011 | Firmicutes | Clostridia | Clostridiales | Ruminococcaceae | Gemmiger |
| Otu322 | 17.37027 | -4.14707 | 1.464642 | -2.83146 | 0.004634 | 0.02756 | Firmicutes | Clostridia | Clostridiales | Ruminococcaceae | Flavonifractor |
| Otu48881 | 3.654532 | 4.227255 | 1.497773 | 2.82236 | 0.004767 | 0.028062 | Verrucomicrobia | Verrucomicrobiae | Verrucomicrobiales | Verrucomicrobiaceae | Akkermansia |
| Otu240 | 9.600241 | 6.508932 | 2.311061 | 2.816426 | 0.004856 | 0.028294 | Firmicutes | Clostridia | Clostridiales | Ruminococcaceae | unclassified |
| Otu147 | 36.40839 | -8.23863 | 2.929821 | -2.81199 | 0.004924 | 0.028398 | Firmicutes | Clostridia | Clostridiales | Ruminococcaceae | Ruminococcus |
| Otu165 | 24.92479 | -7.47849 | 2.668337 | -2.80268 | 0.005068 | 0.028939 | Firmicutes | Clostridia | Clostridiales | Lachnospiraceae | Clostridium XlVa |
| Otu51 | 661.3646 | -2.44086 | 0.875075 | -2.78932 | 0.005282 | 0.029861 | Firmicutes | Clostridia | Clostridiales | Ruminococcaceae | Clostridium IV |
| Otu633 | 4.179616 | -4.11451 | 1.482069 | -2.77619 | 0.0055 | 0.030268 | Firmicutes | Clostridia | Clostridiales | Lachnospiraceae | unclassified |
| Otu8529 | 6.687122 | -3.0016 | 1.081491 | -2.77543 | 0.005513 | 0.030268 | Firmicutes | Clostridia | Clostridiales | Ruminococcaceae | unclassified |
| Otu115746 | 3.753993 | -4.3477 | 1.569174 | -2.77069 | 0.005594 | 0.030419 | Bacteroidetes | Bacteroidia | Bacteroidales | Porphyromonadaceae | Coprobacter |
| Otu349 | 24.92578 | -2.72841 | 0.986582 | -2.76552 | 0.005683 | 0.030614 | Firmicutes | Clostridia | Clostridiales | Lachnospiraceae | unclassified |
| Otu16348 | 6.165824 | -4.60524 | 1.669749 | -2.75804 | 0.005815 | 0.031031 | Bacteroidetes | Bacteroidia | Bacteroidales | Rikenellaceae | Alistipes |
| Otu42820 | 3.90308 | -5.0013 | 1.837016 | -2.72251 | 0.006479 | 0.034253 | Firmicutes | Clostridia | Clostridiales | Ruminococcaceae | Oscillibacter |
| Otu304 | 9.96312 | 5.27723 | 1.976595 | 2.66986 | 0.007588 | 0.039752 | Firmicutes | Clostridia | Clostridiales | Lachnospiraceae | Clostridium XlVa |
| Otu437 | 10.96829 | -3.73206 | 1.399882 | -2.66598 | 0.007676 | 0.039847 | Bacteroidetes | Bacteroidia | Bacteroidales | Rikenellaceae | Alistipes |
| Otu98841 | 12.93969 | -3.46978 | 1.312757 | -2.64313 | 0.008214 | 0.042256 | Bacteroidetes | Bacteroidia | Bacteroidales | Porphyromonadaceae | unclassified |
| Otu378 | 24.00697 | -2.31452 | 0.879233 | -2.63243 | 0.008478 | 0.043221 | Firmicutes | Clostridia | Clostridiales | Lachnospiraceae | Clostridium XlVb |
| Otu24586 | 5.074349 | -4.06411 | 1.557903 | -2.6087 | 0.009089 | 0.045367 | Firmicutes | Clostridia | Clostridiales | Ruminococcaceae | Faecalibacterium |
| Otu30621 | 6.391238 | -4.45632 | 1.708303 | -2.60862 | 0.009091 | 0.045367 | Firmicutes | Clostridia | Clostridiales | Ruminococcaceae | Gemmiger |
| Otu243 | 35.05997 | -3.1334 | 1.201969 | -2.60689 | 0.009137 | 0.045367 | Firmicutes | Clostridia | Clostridiales | Ruminococcaceae | unclassified |
| Otu98782 | 37.2956 | -7.47141 | 2.883596 | -2.591 | 0.00957 | 0.047106 | Verrucomicrobia | Opitutae | Opitutales | Opitutaceae | unclassified |
| Otu70905 | 3.909285 | 4.232839 | 1.638032 | 2.5841 | 0.009763 | 0.047648 | Verrucomicrobia | Verrucomicrobiae | Verrucomicrobiales | Verrucomicrobiaceae | Akkermansia |
| Otu8428 | 2.902242 | 3.530614 | 1.370027 | 2.577039 | 0.009965 | 0.04789 | Firmicutes | Clostridia | Clostridiales | Ruminococcaceae | Clostridium IV |
| Otu481 | 7.5882 | -2.87243 | 1.116381 | -2.57298 | 0.010083 | 0.04789 | Firmicutes | Clostridia | Clostridiales | Lachnospiraceae | unclassified |
| Otu24335 | 9.65395 | -4.01775 | 1.56263 | -2.57114 | 0.010136 | 0.04789 | Firmicutes | Clostridia | Clostridiales | Ruminococcaceae | Cellulosibacter |
| Otu12 | 491.5933 | -2.11133 | 0.821295 | -2.57073 | 0.010148 | 0.04789 | Firmicutes | Clostridia | Clostridiales | Ruminococcaceae | unclassified |

Table S4 The DESeq2 results showing the OTUs associated with sex.

| OUT_ID | baseMean | log2FoldChange | lfcSE | stat | pvalue | padj | Phylum | Class | Order | Family | Genus |
| --- | --- | --- | --- | --- | --- | --- | --- | --- | --- | --- | --- |
| Otu198 | 16.34719 | -23.7392 | 2.951119 | -8.04412 | 8.69E-16 | 5.44E-13 | Bacteroidetes | Bacteroidia | Bacteroidales | Porphyromonadaceae | Odoribacter |
| Otu94 | 12.65058 | -23.3961 | 2.951386 | -7.92714 | 2.24E-15 | 7.02E-13 | Firmicutes | Clostridia | Clostridiales | Lachnospiraceae | Clostridium XlVa |
| Otu244 | 19.5752 | 22.71962 | 2.960824 | 7.673412 | 1.67E-14 | 3.49E-12 | Firmicutes | Clostridia | Clostridiales | Lachnospiraceae | Clostridium XlVb |
| Otu101 | 169.0063 | 5.576982 | 1.165197 | 4.786301 | 1.70E-06 | 0.000266 | Firmicutes | Negativicutes | Selenomonadales | Acidaminococcaceae | Phascolarctobacterium |
| Otu138 | 99.65357 | 5.65579 | 1.312703 | 4.308507 | 1.64E-05 | 0.002058 | Bacteroidetes | Bacteroidia | Bacteroidales | Bacteroidaceae | Bacteroides |
| Otu172 | 23.89482 | -4.81348 | 1.212019 | -3.97145 | 7.14E-05 | 0.007453 | Firmicutes | Clostridia | Clostridiales | Ruminococcaceae | unclassified |
| Otu48 | 28.48522 | 5.868035 | 1.548505 | 3.789484 | 0.000151 | 0.0135 | Firmicutes | Clostridia | Clostridiales | Lachnospiraceae | unclassified |
| Otu134 | 47.41592 | -4.69188 | 1.262775 | -3.71553 | 0.000203 | 0.015128 | Firmicutes | Clostridia | Clostridiales | Ruminococcaceae | Gemmiger |
| Otu86 | 101.5699 | -2.62301 | 0.710808 | -3.69018 | 0.000224 | 0.015128 | Firmicutes | Clostridia | Clostridiales | Lachnospiraceae | Clostridium XlVa |
| Otu22844 | 10.7382 | -4.77328 | 1.300285 | -3.67095 | 0.000242 | 0.015128 | Firmicutes | Clostridia | Clostridiales | Ruminococcaceae | Anaerofilum |
| Otu422 | 8.001526 | -6.57185 | 1.853805 | -3.54506 | 0.000393 | 0.020542 | Firmicutes | Clostridia | Clostridiales | Ruminococcaceae | unclassified |
| Otu31 | 1248.903 | 4.435275 | 1.251413 | 3.544214 | 0.000394 | 0.020542 | Firmicutes | Clostridia | Clostridiales | Lachnospiraceae | Clostridium XlVa |
| Otu66 | 163.9585 | 2.42947 | 0.715795 | 3.394088 | 0.000689 | 0.033158 | Bacteroidetes | Bacteroidia | Bacteroidales | Bacteroidaceae | Bacteroides |
| Otu85 | 384.1566 | 2.518691 | 0.75745 | 3.325224 | 0.000883 | 0.039504 | Bacteroidetes | Bacteroidia | Bacteroidales | Bacteroidaceae | Bacteroides |

Table S5 The DESeq2 results showing the OTUs associated with diet

| \| OUT_ID \| baseMean \| log2FoldChange \| lfcSE \| stat \| pvalue \| padj \| Phylum \| Class \| Order \| Family \| Genus \| \| --- \| --- \| --- \| --- \| --- \| --- \| --- \| --- \| --- \| --- \| --- \| --- \| \| Otu165 \| 24.92479 \| -24.1928 \| 2.574991 \| -9.39529 \| 5.71E-21 \| 3.57E-18 \| Firmicutes \| Clostridia \| Clostridiales \| Lachnospiraceae \| Clostridium XlVa \| \| Otu73 \| 125.6017 \| -10.5269 \| 1.180132 \| -8.92014 \| 4.66E-19 \| 1.46E-16 \| Firmicutes \| Clostridia \| Clostridiales \| Lachnospiraceae \| Clostridium XlVa \| \| Otu52 \| 114.4989 \| -9.90799 \| 1.194852 \| -8.29223 \| 1.11E-16 \| 2.32E-14 \| Firmicutes \| Clostridia \| Clostridiales \| Lachnospiraceae \| unclassified \| \| Otu198 \| 16.34719 \| -23.6163 \| 2.945839 \| -8.01682 \| 1.09E-15 \| 1.70E-13 \| Bacteroidetes \| Bacteroidia \| Bacteroidales \| Porphyromonadaceae \| Odoribacter \| \| Otu94 \| 15.63401 \| 22.52673 \| 2.945755 \| 7.647182 \| 2.05E-14 \| 2.57E-12 \| Firmicutes \| Clostridia \| Clostridiales \| Lachnospiraceae \| Clostridium XlVa \| \| Otu133 \| 77.55505 \| -9.3448 \| 1.278868 \| -7.30708 \| 2.73E-13 \| 2.84E-11 \| Firmicutes \| Clostridia \| Clostridiales \| Ruminococcaceae \| unclassified \| \| Otu30 \| 565.7969 \| -11.0633 \| 1.660554 \| -6.66244 \| 2.69E-11 \| 2.17E-09 \| Firmicutes \| Clostridia \| Clostridiales \| Ruminococcaceae \| Ruminococcus \| \| Otu116 \| 85.01199 \| -7.99315 \| 1.200516 \| -6.6581 \| 2.77E-11 \| 2.17E-09 \| Firmicutes \| Clostridia \| Clostridiales \| Ruminococcaceae \| Flavonifractor \| \| Otu429 \| 14.17863 \| -6.88817 \| 1.043946 \| -6.59821 \| 4.16E-11 \| 2.89E-09 \| Firmicutes \| Clostridia \| Clostridiales \| Lachnospiraceae \| Coprococcus \| \| Otu126 \| 122.7446 \| -10.0069 \| 1.692663 \| -5.91192 \| 3.38E-09 \| 2.11E-07 \| Bacteroidetes \| Bacteroidia \| Bacteroidales \| Prevotellaceae \| Prevotella \| \| Otu121 \| 82.56386 \| -4.46072 \| 0.768825 \| -5.80201 \| 6.55E-09 \| 3.72E-07 \| Firmicutes \| Clostridia \| Clostridiales \| Ruminococcaceae \| unclassified \| \| Otu149 \| 50.56541 \| -8.46898 \| 1.476009 \| -5.73776 \| 9.59E-09 \| 5.00E-07 \| Firmicutes \| Clostridia \| Clostridiales \| Ruminococcaceae \| Faecalibacterium \| \| Otu332 \| 17.62821 \| -4.37889 \| 0.805566 \| -5.43579 \| 5.46E-08 \| 2.62E-06 \| Firmicutes \| Clostridia \| Clostridiales \| Ruminococcaceae \| unclassified \| \| Otu87 \| 58.64355 \| -9.18696 \| 1.731402 \| -5.30608 \| 1.12E-07 \| 5.00E-06 \| Bacteroidetes \| Bacteroidia \| Bacteroidales \| Rikenellaceae \| Alistipes \| \| Otu251 \| 21.68091 \| -7.99334 \| 1.511355 \| -5.28885 \| 1.23E-07 \| 5.13E-06 \| Firmicutes \| Clostridia \| Clostridiales \| Ruminococcaceae \| Ruminococcus \| \| Otu117 \| 63.66358 \| -4.66265 \| 0.888764 \| -5.24622 \| 1.55E-07 \| 6.06E-06 \| Firmicutes \| Erysipelotrichia \| Erysipelotrichales \| Erysipelotrichaceae \| unclassified \| \| Otu256 \| 7.984642 \| -6.30475 \| 1.20707 \| -5.22319 \| 1.76E-07 \| 6.47E-06 \| Firmicutes \| Clostridia \| Clostridiales \| Lachnospiraceae \| unclassified \| \| Otu381 \| 13.53454 \| -5.80487 \| 1.12545 \| -5.15782 \| 2.50E-07 \| 8.68E-06 \| Firmicutes \| Clostridia \| Clostridiales \| Lachnospiraceae \| unclassified \| \| Otu1 \| 58.51526 \| 6.174478 \| 1.229473 \| 5.022053 \| 5.11E-07 \| 1.68E-05 \| Firmicutes \| Bacilli \| Lactobacillales \| Lactobacillaceae \| Lactobacillus \| \| Otu61 \| 278.5681 \| -4.5727 \| 0.926559 \| -4.93514 \| 8.01E-07 \| 2.50E-05 \| Bacteroidetes \| Bacteroidia \| Bacteroidales \| Rikenellaceae \| Alistipes \| \| Otu177 \| 51.30854 \| -5.37146 \| 1.122962 \| -4.7833 \| 1.72E-06 \| 5.13E-05 \| Firmicutes \| Clostridia \| Clostridiales \| Ruminococcaceae \| Flavonifractor \| \| Otu45 \| 157.6673 \| -3.6599 \| 0.766874 \| -4.7725 \| 1.82E-06 \| 5.17E-05 \| Firmicutes \| Clostridia \| Clostridiales \| Lachnospiraceae \| unclassified \| \| Otu78 \| 112.4839 \| -9.33006 \| 2.150426 \| -4.3387 \| 1.43E-05 \| 0.000389 \| Firmicutes \| Negativicutes \| Selenomonadales \| Acidaminococcaceae \| Phascolarctobacterium \| \| Otu263 \| 21.41545 \| -5.388 \| 1.252062 \| -4.3033 \| 1.68E-05 \| 0.000438 \| Firmicutes \| Clostridia \| Clostridiales \| Ruminococcaceae \| Flavonifractor \| \| Otu261 \| 13.61724 \| 7.098793 \| 1.683361 \| 4.217035 \| 2.48E-05 \| 0.000611 \| Firmicutes \| Clostridia \| Clostridiales \| Ruminococcaceae \| Sporobacter \| \| Otu24579 \| 9.027956 \| -6.72723 \| 1.597557 \| -4.21095 \| 2.54E-05 \| 0.000611 \| Firmicutes \| Clostridia \| Clostridiales \| Ruminococcaceae \| Gemmiger \| \| Otu200 \| 49.97178 \| -3.93078 \| 0.937038 \| -4.1949 \| 2.73E-05 \| 0.000632 \| Firmicutes \| Clostridia \| Clostridiales \| Ruminococcaceae \| unclassified \| \| Otu59 \| 259.0558 \| -3.19964 \| 0.768762 \| -4.16207 \| 3.15E-05 \| 0.000704 \| Bacteroidetes \| Bacteroidia \| Bacteroidales \| Porphyromonadaceae \| Odoribacter \| \| Otu19 \| 806.359 \| -3.66814 \| 0.901128 \| -4.0706 \| 4.69E-05 \| 0.001011 \| Bacteroidetes \| Bacteroidia \| Bacteroidales \| Rikenellaceae \| Alistipes \| \| Otu0 \| 7001.999 \| 3.103955 \| 0.774506 \| 4.007658 \| 6.13E-05 \| 0.001278 \| Verrucomicrobia \| Verrucomicrobiae \| Verrucomicrobiales \| Verrucomicrobiaceae \| Akkermansia \| \| Otu97 \| 245.5272 \| -2.47334 \| 0.633398 \| -3.90487 \| 9.43E-05 \| 0.001901 \| Firmicutes \| Clostridia \| Clostridiales \| Ruminococcaceae \| Gemmiger \| \| Otu203 \| 19.30728 \| -5.16667 \| 1.362172 \| -3.79296 \| 0.000149 \| 0.002907 \| Euryarchaeota \| Thermoplasmata \| Methanomassiliicoccales \| Methanomassiliicoccaceae \| Methanomassiliicoccus \| \| Otu16439 \| 7.625106 \| -6.23548 \| 1.647725 \| -3.7843 \| 0.000154 \| 0.002919 \| Firmicutes \| Clostridia \| Clostridiales \| Ruminococcaceae \| Gemmiger \| \| Otu393 \| 17.29644 \| -2.70448 \| 0.717375 \| -3.76997 \| 0.000163 \| 0.003001 \| Actinobacteria \| Actinobacteria \| Coriobacteriales \| Coriobacteriaceae \| Adlercreutzia \| \| Otu1403 \| 5.35428 \| -5.20057 \| 1.390389 \| -3.74037 \| 0.000184 \| 0.003281 \| Firmicutes \| Clostridia \| Clostridiales \| Lachnospiraceae \| unclassified \| \| Otu4 \| 9.050463 \| 4.792125 \| 1.293974 \| 3.703418 \| 0.000213 \| 0.003635 \| Proteobacteria \| Gammaproteobacteria \| Pseudomonadales \| Pseudomonadaceae \| Pseudomonas \| \| Otu299 \| 13.71311 \| -3.19537 \| 0.865009 \| -3.69403 \| 0.000221 \| 0.003635 \| Firmicutes \| Clostridia \| Clostridiales \| Lachnospiraceae \| Lachnospiracea_incertae_sedis \| \| Otu124 \| 52.75908 \| -5.13084 \| 1.389989 \| -3.69128 \| 0.000223 \| 0.003635 \| Proteobacteria \| Deltaproteobacteria \| Desulfovibrionales \| Desulfovibrionaceae \| unclassified \| \| Otu143 \| 26.38648 \| -5.80053 \| 1.573199 \| -3.68709 \| 0.000227 \| 0.003635 \| Bacteroidetes \| Bacteroidia \| Bacteroidales \| Prevotellaceae \| Paraprevotella \| \| Otu195 \| 61.00863 \| 8.901418 \| 2.486588 \| 3.579772 \| 0.000344 \| 0.005308 \| Firmicutes \| Clostridia \| Clostridiales \| Ruminococcaceae \| unclassified \| \| Otu422 \| 8.001526 \| -6.55558 \| 1.832957 \| -3.57651 \| 0.000348 \| 0.005308 \| Firmicutes \| Clostridia \| Clostridiales \| Ruminococcaceae \| unclassified \| \| Otu239 \| 26.96614 \| 6.851954 \| 1.922639 \| 3.563827 \| 0.000365 \| 0.005439 \| Firmicutes \| Clostridia \| Clostridiales \| Lachnospiraceae \| unclassified \| \| Otu16 \| 603.6093 \| -2.13623 \| 0.602491 \| -3.54566 \| 0.000392 \| 0.005692 \| Firmicutes \| Clostridia \| Clostridiales \| Ruminococcaceae \| Gemmiger \| \| Otu11 \| 585.6318 \| -2.3235 \| 0.667194 \| -3.48249 \| 0.000497 \| 0.006732 \| Firmicutes \| Clostridia \| Clostridiales \| Ruminococcaceae \| unclassified \| \| Otu139 \| 112.7042 \| -2.81545 \| 0.80892 \| -3.4805 \| 0.0005 \| 0.006732 \| Firmicutes \| Clostridia \| Clostridiales \| Ruminococcaceae \| Clostridium IV \| \| Otu13 \| 52.33512 \| 10.24321 \| 2.945125 \| 3.478022 \| 0.000505 \| 0.006732 \| Firmicutes \| Clostridia \| Clostridiales \| Ruminococcaceae \| Ruminococcus \| \| Otu254 \| 6.104219 \| -5.12447 \| 1.473647 \| -3.47741 \| 0.000506 \| 0.006732 \| Firmicutes \| Clostridia \| Clostridiales \| Lachnospiraceae \| Clostridium XlVa \| \| Otu396 \| 4.379653 \| -5.43541 \| 1.589723 \| -3.41909 \| 0.000628 \| 0.008181 \| Firmicutes \| Clostridia \| Clostridiales \| Lachnospiraceae \| Clostridium XlVa \| \| Otu470 \| 7.022147 \| -6.36728 \| 1.869461 \| -3.40595 \| 0.000659 \| 0.008399 \| Proteobacteria \| Deltaproteobacteria \| Bdellovibrionales \| Bdellovibrionaceae \| Vampirovibrio \| \| Otu178 \| 74.30913 \| -2.75124 \| 0.808999 \| -3.4008 \| 0.000672 \| 0.008399 \| Firmicutes \| Clostridia \| Clostridiales \| Lachnospiraceae \| Clostridium XlVb \| \| Otu604 \| 10.38218 \| -3.52432 \| 1.041385 \| -3.38426 \| 0.000714 \| 0.008746 \| Firmicutes \| Clostridia \| Clostridiales \| Ruminococcaceae \| unclassified \| \| Otu461 \| 4.365728 \| 5.457843 \| 1.624734 \| 3.359223 \| 0.000782 \| 0.009394 \| Firmicutes \| Erysipelotrichia \| Erysipelotrichales \| Erysipelotrichaceae \| Clostridium XVIII \| \| Otu48 \| 28.48522 \| 5.052612 \| 1.508397 \| 3.349657 \| 0.000809 \| 0.009541 \| Firmicutes \| Clostridia \| Clostridiales \| Lachnospiraceae \| unclassified \| \| Otu220 \| 23.6307 \| -6.9222 \| 2.078538 \| -3.33032 \| 0.000867 \| 0.01004 \| Firmicutes \| Erysipelotrichia \| Erysipelotrichales \| Erysipelotrichaceae \| Clostridium XVIII \| \| Otu16348 \| 6.165824 \| -5.92763 \| 1.804322 \| -3.28524 \| 0.001019 \| 0.011579 \| Bacteroidetes \| Bacteroidia \| Bacteroidales \| Rikenellaceae \| Alistipes \| \| Otu351 \| 18.29052 \| -3.12433 \| 0.957472 \| -3.2631 \| 0.001102 \| 0.012299 \| Firmicutes \| Clostridia \| Clostridiales \| Ruminococcaceae \| Clostridium IV \| \| Otu326 \| 10.85104 \| -4.96093 \| 1.531761 \| -3.23871 \| 0.001201 \| 0.013166 \| Tenericutes \| Mollicutes \| Anaeroplasmatales \| Anaeroplasmataceae \| Anaeroplasma \| \| Otu22 \| 1023.655 \| -2.56513 \| 0.80038 \| -3.20489 \| 0.001351 \| 0.01456 \| Firmicutes \| Clostridia \| Clostridiales \| Lachnospiraceae \| Ruminococcus2 \| \| Otu222 \| 20.18433 \| -4.5104 \| 1.412274 \| -3.19372 \| 0.001405 \| 0.014879 \| Bacteroidetes \| Bacteroidia \| Bacteroidales \| Rikenellaceae \| Alistipes \| \| Otu359 \| 18.29835 \| -4.81346 \| 1.510955 \| -3.18571 \| 0.001444 \| 0.015042 \| Bacteroidetes \| Bacteroidia \| Bacteroidales \| Rikenellaceae \| Alistipes \| \| Otu218 \| 4.746243 \| -5.79913 \| 1.826187 \| -3.17554 \| 0.001496 \| 0.015323 \| Proteobacteria \| Alphaproteobacteria \| Rhodospirillales \| Rhodospirillaceae \| unclassified \| \| Otu463 \| 7.85337 \| -4.07729 \| 1.288345 \| -3.16475 \| 0.001552 \| 0.015647 \| Firmicutes \| Clostridia \| Clostridiales \| Lachnospiraceae \| Clostridium XlVb \| \| Otu105 \| 118.8511 \| -6.30365 \| 2.032742 \| -3.10106 \| 0.001928 \| 0.01913 \| Bacteroidetes \| Bacteroidia \| Bacteroidales \| Bacteroidaceae \| Bacteroides \| \| Otu104 \| 84.38752 \| -3.43313 \| 1.112476 \| -3.08603 \| 0.002028 \| 0.019434 \| Firmicutes \| Clostridia \| Clostridiales \| Lachnospiraceae \| Clostridium XlVa \| \| Otu48881 \| 3.654532 \| 4.94181 \| 1.602045 \| 3.084688 \| 0.002038 \| 0.019434 \| Verrucomicrobia \| Verrucomicrobiae \| Verrucomicrobiales \| Verrucomicrobiaceae \| Akkermansia \| \| Otu8378 \| 2.814489 \| 4.818465 \| 1.563131 \| 3.082572 \| 0.002052 \| 0.019434 \| Firmicutes \| Clostridia \| Clostridiales \| Ruminococcaceae \| unclassified \| \| Otu55 \| 611.5629 \| -3.00428 \| 0.983785 \| -3.0538 \| 0.00226 \| 0.021078 \| Firmicutes \| Clostridia \| Clostridiales \| Ruminococcaceae \| Subdoligranulum \| \| Otu112 \| 43.67923 \| 7.403152 \| 2.474302 \| 2.992016 \| 0.002771 \| 0.025473 \| Bacteroidetes \| Bacteroidia \| Bacteroidales \| Bacteroidaceae \| Bacteroides \| \| Otu32 \| 1559.438 \| -2.1322 \| 0.7163 \| -2.97668 \| 0.002914 \| 0.026357 \| Firmicutes \| Clostridia \| Clostridiales \| Ruminococcaceae \| Oscillibacter \| \| Otu17068 \| 4.668094 \| -5.7754 \| 1.943799 \| -2.97119 \| 0.002966 \| 0.026357 \| Firmicutes \| Clostridia \| Clostridiales \| Ruminococcaceae \| unclassified \| \| Otu631 \| 4.8605 \| -4.78532 \| 1.612122 \| -2.96833 \| 0.002994 \| 0.026357 \| Firmicutes \| Clostridia \| Clostridiales \| Ruminococcaceae \| unclassified \| \| Otu98 \| 10.67224 \| 4.845701 \| 1.635118 \| 2.963517 \| 0.003041 \| 0.026401 \| Firmicutes \| Clostridia \| Clostridiales \| Ruminococcaceae \| Clostridium IV \| \| Otu334 \| 18.18618 \| -3.4147 \| 1.156596 \| -2.95237 \| 0.003153 \| 0.026999 \| Firmicutes \| Clostridia \| Clostridiales \| Lachnospiraceae \| unclassified \| \| Otu408 \| 21.12904 \| -2.93378 \| 0.996877 \| -2.94297 \| 0.003251 \| 0.027456 \| Firmicutes \| Clostridia \| Clostridiales \| Lachnospiraceae \| unclassified \| \| Otu39544 \| 4.07383 \| 5.35441 \| 1.841225 \| 2.90807 \| 0.003637 \| 0.030306 \| Verrucomicrobia \| Verrucomicrobiae \| Verrucomicrobiales \| Verrucomicrobiaceae \| Akkermansia \| \| Otu42820 \| 3.90308 \| -5.26921 \| 1.821486 \| -2.89281 \| 0.003818 \| 0.031399 \| Firmicutes \| Clostridia \| Clostridiales \| Ruminococcaceae \| Oscillibacter \| \| Otu232 \| 8.649617 \| -8.46635 \| 2.946717 \| -2.87315 \| 0.004064 \| 0.032987 \| Firmicutes \| Clostridia \| Clostridiales \| Ruminococcaceae \| unclassified \| \| Otu295 \| 12.53164 \| -3.72591 \| 1.307081 \| -2.85056 \| 0.004364 \| 0.03497 \| Firmicutes \| Clostridia \| Clostridiales \| Ruminococcaceae \| unclassified \| \| Otu115746 \| 3.753993 \| -4.40491 \| 1.558892 \| -2.82567 \| 0.004718 \| 0.037328 \| Bacteroidetes \| Bacteroidia \| Bacteroidales \| Porphyromonadaceae \| Coprobacter \| \| Otu322 \| 17.37027 \| -4.05856 \| 1.451722 \| -2.79569 \| 0.005179 \| 0.040461 \| Firmicutes \| Clostridia \| Clostridiales \| Ruminococcaceae \| Flavonifractor \| \| Otu8347 \| 7.453675 \| -6.45258 \| 2.34452 \| -2.7522 \| 0.00592 \| 0.045677 \| Firmicutes \| Clostridia \| Clostridiales \| Lachnospiraceae \| unclassified \| \| Otu542 \| 2.26227 \| -4.73114 \| 1.732999 \| -2.73003 \| 0.006333 \| 0.048268 \| Bacteroidetes \| Bacteroidia \| Bacteroidales \| Rikenellaceae \| Alistipes \| \| Otu321 \| 12.38491 \| -6.93948 \| 2.548462 \| -2.72301 \| 0.006469 \| 0.048713 \| Proteobacteria \| Deltaproteobacteria \| Desulfovibrionales \| Desulfovibrionaceae \| Desulfovibrio \| \| Otu70905 \| 3.909285 \| 4.525959 \| 1.666518 \| 2.715817 \| 0.006611 \| 0.049191 \| Verrucomicrobia \| Verrucomicrobiae \| Verrucomicrobiales \| Verrucomicrobiaceae \| Akkermansia \| |  |  |  |  |  |  |  |  |  |  |  |
| --- | --- | --- | --- | --- | --- | --- | --- | --- | --- | --- | --- | --- | --- | --- | --- | --- | --- | --- | --- | --- | --- | --- | --- | --- | --- | --- | --- | --- | --- | --- | --- | --- | --- | --- | --- | --- | --- | --- | --- | --- | --- | --- | --- | --- | --- | --- | --- | --- | --- | --- | --- | --- | --- | --- | --- | --- | --- | --- | --- | --- | --- | --- | --- | --- | --- | --- | --- | --- | --- | --- | --- | --- | --- | --- | --- | --- | --- | --- | --- | --- | --- | --- | --- | --- | --- | --- | --- | --- | --- | --- | --- | --- | --- | --- | --- | --- | --- | --- | --- | --- | --- | --- | --- | --- | --- | --- | --- | --- | --- | --- | --- | --- | --- | --- | --- | --- | --- | --- | --- | --- | --- | --- | --- | --- | --- | --- | --- | --- | --- | --- | --- | --- | --- | --- | --- | --- | --- | --- | --- | --- | --- | --- | --- | --- | --- | --- | --- | --- | --- | --- | --- | --- | --- | --- | --- | --- | --- | --- | --- | --- | --- | --- | --- | --- | --- | --- | --- | --- | --- | --- | --- | --- | --- | --- | --- | --- | --- | --- | --- | --- | --- | --- | --- | --- | --- | --- | --- | --- | --- | --- | --- | --- | --- | --- | --- | --- | --- | --- | --- | --- | --- | --- | --- | --- | --- | --- | --- | --- | --- | --- | --- | --- | --- | --- | --- | --- | --- | --- | --- | --- | --- | --- | --- | --- | --- | --- | --- | --- | --- | --- | --- | --- | --- | --- | --- | --- | --- | --- | --- | --- | --- | --- | --- | --- | --- | --- | --- | --- | --- | --- | --- | --- | --- | --- | --- | --- | --- | --- | --- | --- | --- | --- | --- | --- | --- | --- | --- | --- | --- | --- | --- | --- | --- | --- | --- | --- | --- | --- | --- | --- | --- | --- | --- | --- | --- | --- | --- | --- | --- | --- | --- | --- | --- | --- | --- | --- | --- | --- | --- | --- | --- | --- | --- | --- | --- | --- | --- | --- | --- | --- | --- | --- | --- | --- | --- | --- | --- | --- | --- | --- | --- | --- | --- | --- | --- | --- | --- | --- | --- | --- | --- | --- | --- | --- | --- | --- | --- | --- | --- | --- | --- | --- | --- | --- | --- | --- | --- | --- | --- | --- | --- | --- | --- | --- | --- | --- | --- | --- | --- | --- | --- | --- | --- | --- | --- | --- | --- | --- | --- | --- | --- | --- | --- | --- | --- | --- | --- | --- | --- | --- | --- | --- | --- | --- | --- | --- | --- | --- | --- | --- | --- | --- | --- | --- | --- | --- | --- | --- | --- | --- | --- | --- | --- | --- | --- | --- | --- | --- | --- | --- | --- | --- | --- | --- | --- | --- | --- | --- | --- | --- | --- | --- | --- | --- | --- | --- | --- | --- | --- | --- | --- | --- | --- | --- | --- | --- | --- | --- | --- | --- | --- | --- | --- | --- | --- | --- | --- | --- | --- | --- | --- | --- | --- | --- | --- | --- | --- | --- | --- | --- | --- | --- | --- | --- | --- | --- | --- | --- | --- | --- | --- | --- | --- | --- | --- | --- | --- | --- | --- | --- | --- | --- | --- | --- | --- | --- | --- | --- | --- | --- | --- | --- | --- | --- | --- | --- | --- | --- | --- | --- | --- | --- | --- | --- | --- | --- | --- | --- | --- | --- | --- | --- | --- | --- | --- | --- | --- | --- | --- | --- | --- | --- | --- | --- | --- | --- | --- | --- | --- | --- | --- | --- | --- | --- | --- | --- | --- | --- | --- | --- | --- | --- | --- | --- | --- | --- | --- | --- | --- | --- | --- | --- | --- | --- | --- | --- | --- | --- | --- | --- | --- | --- | --- | --- | --- | --- | --- | --- | --- | --- | --- | --- | --- | --- | --- | --- | --- | --- | --- | --- | --- | --- | --- | --- | --- | --- | --- | --- | --- | --- | --- | --- | --- | --- | --- | --- | --- | --- | --- | --- | --- | --- | --- | --- | --- | --- | --- | --- | --- | --- | --- | --- | --- | --- | --- | --- | --- | --- | --- | --- | --- | --- | --- | --- | --- | --- | --- | --- | --- | --- | --- | --- | --- | --- | --- | --- | --- | --- | --- | --- | --- | --- | --- | --- | --- | --- | --- | --- | --- | --- | --- | --- | --- | --- | --- | --- | --- | --- | --- | --- | --- | --- | --- | --- | --- | --- | --- | --- | --- | --- | --- | --- | --- | --- | --- | --- | --- | --- | --- | --- | --- | --- | --- | --- | --- | --- | --- | --- | --- | --- | --- | --- | --- | --- | --- | --- | --- | --- | --- | --- | --- | --- | --- | --- | --- | --- | --- | --- | --- | --- | --- | --- | --- | --- | --- | --- | --- | --- | --- | --- | --- | --- | --- | --- | --- | --- | --- | --- | --- | --- | --- | --- | --- | --- | --- | --- | --- | --- | --- | --- | --- | --- | --- | --- | --- | --- | --- | --- | --- | --- | --- | --- | --- | --- | --- | --- | --- | --- | --- | --- | --- | --- | --- | --- | --- | --- | --- | --- | --- | --- | --- | --- | --- | --- | --- | --- | --- | --- | --- | --- | --- | --- | --- | --- | --- | --- | --- | --- | --- | --- | --- | --- | --- | --- | --- | --- | --- | --- | --- | --- | --- | --- | --- | --- | --- | --- | --- | --- | --- | --- | --- | --- | --- | --- | --- | --- | --- | --- | --- | --- | --- | --- | --- | --- | --- | --- | --- | --- | --- | --- | --- | --- | --- | --- | --- | --- | --- | --- | --- | --- | --- | --- | --- | --- | --- | --- | --- | --- | --- | --- | --- | --- | --- | --- | --- | --- | --- | --- | --- | --- | --- | --- | --- | --- | --- | --- | --- | --- | --- | --- | --- | --- | --- | --- | --- | --- | --- | --- | --- | --- | --- | --- | --- | --- | --- | --- | --- | --- | --- | --- | --- | --- | --- | --- | --- | --- | --- | --- | --- | --- | --- | --- | --- | --- | --- | --- | --- | --- | --- | --- | --- | --- | --- | --- | --- | --- | --- | --- | --- | --- | --- | --- | --- | --- | --- | --- | --- | --- | --- | --- | --- | --- | --- | --- | --- | --- | --- | --- | --- | --- | --- | --- | --- | --- | --- | --- | --- | --- | --- | --- | --- | --- | --- | --- | --- | --- | --- | --- | --- | --- | --- | --- | --- | --- | --- | --- | --- | --- | --- | --- | --- | --- | --- | --- | --- | --- | --- | --- | --- | --- | --- | --- | --- | --- | --- | --- | --- | --- | --- | --- | --- | --- | --- | --- | --- | --- | --- | --- | --- | --- | --- | --- | --- | --- | --- | --- | --- | --- | --- | --- | --- | --- | --- | --- | --- | --- | --- | --- | --- | --- | --- | --- | --- | --- | --- | --- | --- | --- | --- | --- | --- |
|  |  |  |  |  |  |  |  |  |  |  |  |
|  |  |  |  |  |  |  |  |  |  |  |  |
|  |  |  |  |  |  |  |  |  |  |  |  |
|  |  |  |  |  |  |  |  |  |  |  |  |
|  |  |  |  |  |  |  |  |  |  |  |  |
|  |  |  |  |  |  |  |  |  |  |  |  |
|  |  |  |  |  |  |  |  |  |  |  |  |
|  |  |  |  |  |  |  |  |  |  |  |  |
|  |  |  |  |  |  |  |  |  |  |  |  |
|  |  |  |  |  |  |  |  |  |  |  |  |
|  |  |  |  |  |  |  |  |  |  |  |  |
|  |  |  |  |  |  |  |  |  |  |  |  |
|  |  |  |  |  |  |  |  |  |  |  |  |
|  |  |  |  |  |  |  |  |  |  |  |  |
|  |  |  |  |  |  |  |  |  |  |  |  |
|  |  |  |  |  |  |  |  |  |  |  |  |
|  |  |  |  |  |  |  |  |  |  |  |  |
|  |  |  |  |  |  |  |  |  |  |  |  |
|  |  |  |  |  |  |  |  |  |  |  |  |
|  |  |  |  |  |  |  |  |  |  |  |  |
|  |  |  |  |  |  |  |  |  |  |  |  |
|  |  |  |  |  |  |  |  |  |  |  |  |
|  |  |  |  |  |  |  |  |  |  |  |  |
|  |  |  |  |  |  |  |  |  |  |  |  |
|  |  |  |  |  |  |  |  |  |  |  |  |
|  |  |  |  |  |  |  |  |  |  |  |  |
|  |  |  |  |  |  |  |  |  |  |  |  |
|  |  |  |  |  |  |  |  |  |  |  |  |
|  |  |  |  |  |  |  |  |  |  |  |  |
|  |  |  |  |  |  |  |  |  |  |  |  |
|  |  |  |  |  |  |  |  |  |  |  |  |
|  |  |  |  |  |  |  |  |  |  |  |  |
|  |  |  |  |  |  |  |  |  |  |  |  |
|  |  |  |  |  |  |  |  |  |  |  |  |
|  |  |  |  |  |  |  |  |  |  |  |  |
|  |  |  |  |  |  |  |  |  |  |  |  |
|  |  |  |  |  |  |  |  |  |  |  |  |
|  |  |  |  |  |  |  |  |  |  |  |  |
|  |  |  |  |  |  |  |  |  |  |  |  |
|  |  |  |  |  |  |  |  |  |  |  |  |
|  |  |  |  |  |  |  |  |  |  |  |  |
|  |  |  |  |  |  |  |  |  |  |  |  |
|  |  |  |  |  |  |  |  |  |  |  |  |
|  |  |  |  |  |  |  |  |  |  |  |  |
|  |  |  |  |  |  |  |  |  |  |  |  |
|  |  |  |  |  |  |  |  |  |  |  |  |
|  |  |  |  |  |  |  |  |  |  |  |  |
|  |  |  |  |  |  |  |  |  |  |  |  |
|  |  |  |  |  |  |  |  |  |  |  |  |
|  |  |  |  |  |  |  |  |  |  |  |  |
|  |  |  |  |  |  |  |  |  |  |  |  |
|  |  |  |  |  |  |  |  |  |  |  |  |
|  |  |  |  |  |  |  |  |  |  |  |  |
|  |  |  |  |  |  |  |  |  |  |  |  |
|  |  |  |  |  |  |  |  |  |  |  |  |
|  |  |  |  |  |  |  |  |  |  |  |  |
|  |  |  |  |  |  |  |  |  |  |  |  |
|  |  |  |  |  |  |  |  |  |  |  |  |
|  |  |  |  |  |  |  |  |  |  |  |  |
|  |  |  |  |  |  |  |  |  |  |  |  |
|  |  |  |  |  |  |  |  |  |  |  |  |
|  |  |  |  |  |  |  |  |  |  |  |  |
|  |  |  |  |  |  |  |  |  |  |  |  |
|  |  |  |  |  |  |  |  |  |  |  |  |
|  |  |  |  |  |  |  |  |  |  |  |  |
|  |  |  |  |  |  |  |  |  |  |  |  |
|  |  |  |  |  |  |  |  |  |  |  |  |
|  |  |  |  |  |  |  |  |  |  |  |  |
|  |  |  |  |  |  |  |  |  |  |  |  |
|  |  |  |  |  |  |  |  |  |  |  |  |
|  |  |  |  |  |  |  |  |  |  |  |  |
|  |  |  |  |  |  |  |  |  |  |  |  |
|  |  |  |  |  |  |  |  |  |  |  |  |
|  |  |  |  |  |  |  |  |  |  |  |  |
|  |  |  |  |  |  |  |  |  |  |  |  |
|  |  |  |  |  |  |  |  |  |  |  |  |
|  |  |  |  |  |  |  |  |  |  |  |  |
|  |  |  |  |  |  |  |  |  |  |  |  |
|  |  |  |  |  |  |  |  |  |  |  |  |
|  |  |  |  |  |  |  |  |  |  |  |  |
|  |  |  |  |  |  |  |  |  |  |  |  |
|  |  |  |  |  |  |  |  |  |  |  |  |
|  |  |  |  |  |  |  |  |  |  |  |  |
|  |  |  |  |  |  |  |  |  |  |  |  |
